# A rapid yeast-based reverse genetics system reveals SARS-CoV-2 Omicron BA.2.86 variant spreads faster than Omicron JN.1 variant in primary human nasal and bronchial epithelial airway cultures

**DOI:** 10.1128/mbio.00122-26

**Published:** 2026-05-12

**Authors:** Jiayu Xu, Michelle Chamblee, Cheng Chih Hsu, Fei Jiang, Phylip Chen, Yuexiu Zhang, Xueya Liang, Amal O. Amer, Prosper N. Boyaka, Estelle Cormet-Boyaka, Mark E. Peeples, Jianrong Li

**Affiliations:** 1 Department of Veterinary Biosciences, The Ohio State University 198563 , Columbus, Ohio, USA; 2 Center for Microbe and Immunity Research, Abigail Wexner Research Institute, Nationwide Children’s Hospital 51711, Columbus, Ohio, USA; 3 Department of Microbial Infection and Immunity, The Ohio State University 683676 , Columbus, Ohio, USA; 4 Department of Pediatrics, College of Medicine, The Ohio State University 12305, Columbus, Ohio, USA; 5 Infectious Disease Institute, The Ohio State University 2647, Columbus, Ohio, USA; 6 Center for RNA Biology, The Ohio State University 2647, Columbus, Ohio, USA; Duke University School of Medicine, Durham, North Carolina, USA

**Keywords:** SARS-CoV-2, rapid reverse genetics system, virus spread

## Abstract

Since the COVID-19 pandemic, several reverse genetics platforms for SARS-CoV-2 have been established. In general, a plasmid-based reverse genetics system is stable and easy to manipulate, distribute, and store. However, traditional methods for the assembly of a large viral genome in a plasmid rely on natural and artificially engineered restriction sites, which are inefficient, time-consuming, labor-intensive, and frequently not successful. Here, we developed a yeast-based homologous recombination system that allows the assembly of the SARS-CoV-2 genome as a cDNA in a bacterial artificial chromosome (BAC) plasmid in a single step. The entire protocol from cDNA construction to virus rescue is simple, rapid, accurate, highly efficient, and can be completed in 2 weeks. Using this system, we have quickly generated recombinant SARS-CoV-2 (rSARS-CoV-2) WA1, Omicron BA.2.86, and Omicron JN.1 viruses expressing mCherry, green fluorescent protein (GFP), and NanoLuc luciferase (Nluc) reporters. Insertion of these reporter genes does not significantly alter the replication of SARS-CoV-2 in cell culture. We also compared the replication kinetics of rSARS-CoV-2-WA1, BA.2.86, and JN.1 reporter viruses in *ex vivo* primary human nasal epithelial (HNE) and human bronchial epithelial (HBE) cultures. Omicron BA.2.86 replicated and spread more efficiently than JN.1, which spread much faster than SARS-CoV-2 WA1 in these cultures. In summary, we have developed a highly efficient yeast-based recombinant system for the construction of infectious cDNA clones of SARS-CoV-2, enabling rapid genetic manipulation of SARS-CoV-2. In addition, the reporter viruses generated in this study will be useful for monitoring SARS-CoV-2 infection *in vitro* and *in vivo*.

**IMPORTANCE:**

Reverse genetics systems are an essential tool for probing the biology of viruses, testing antivirals, and developing live-attenuated vaccines. However, it has been a challenge to generate a rapid reverse genetics system for coronaviruses. Here, we developed a rapid, highly efficient reverse genetics system for SARS-CoV-2 that uses yeast homologous recombination. In this procedure, overlapping DNA fragments encompassing the entire SARS-CoV-2 and BAC plasmid fragments containing a yeast replication origin were mixed and transformed into yeast cells to assemble infectious cDNA clones in a single step. This system has enabled us to rapidly generate nine SARS-CoV-2 viruses: WA1, Omicron BA.2.86, and JN.1 viruses each expressing one of three reporters for tracking virus infection *in vitro* and *in vivo*. This method is easy, convenient, and highly efficient, generating infectious cDNA clones within 2 weeks. This system could readily be adapted to construct infectious cDNA clones for other large RNA viruses.

## INTRODUCTION

Coronaviruses (CoVs) are enveloped, single-strand positive-sense RNA viruses, belonging to the order *Nidovirales*. A broad spectrum of CoVs impacts human and animal health. Human CoVs can manifest as seasonal mild respiratory infections caused by strains, such as 229E, NL63, OC43, and HKU1, as well as more severe and fatal diseases such as those induced by severe acute respiratory syndrome coronavirus (SARS-CoV) and Middle East respiratory syndrome coronavirus (MERS-CoV) (1). The emergence of SARS-CoV-2 in late 2019 marked the beginning of a global pandemic that profoundly affected the global economy and public health. Prevention of SARS-CoV-2 has been particularly difficult because of its rapid spread and the frequent emergence of novel variants of concern (VoCs). Since the pandemic began, numerous VoCs have emerged, including the immunogenically diverse Alpha (B.1.1.7), Beta (B.1.351), Gamma (P.1), Delta (B.1.617.2), and Omicron variants and their subvariants (2–5). Although the World Health Organization (WHO) declared the end of the pandemic phase of COVID‐19 on May 5, 2023, the SARS-CoV-2 Omicron variant has continued to evolve rapidly, causing waves of breakthrough infection (5).

In July 2023, the emergence of the Omicron BA.2.86 variant was a turning point in the SARS-CoV-2 evolution (6, 7). The spike protein of BA.2.86 is characterized by more than 30 mutations relative to its ancestral variant BA.2 and 35 mutations relative to the previous dominant Omicron XBB.1.5. Interestingly, Omicron BA.2.86 did not become a dominant virus. Instead, Omicron subvariant JN.1, which only differs by a single amino acid (L455S) from the spike of BA.2.86, became dominant worldwide from late 2023 to May 2024 (8, 9). The L455S mutation reduced the binding affinity of JN.1 for human ACE2 but increased immune evasion, neutralizing antibody escape, and viral transmission (8–14). Since May 2024, several JN.1-derived variants, including JN.1.16, KP.2, KP3, KP3.1.1, XEC, and NB.1.8.1, have been circulating (5, 15). Currently, the Omicron XFG variant is the dominant strain worldwide.

Reverse genetics of RNA viruses is based on the ability to rescue infectivity entirely from cloned full-length viral cDNA. This requirement also provides a major benefit, allowing manipulation of the viral genomic DNA with targeted mutations, deletions, or insertions. It has become an essential tool for studying the viral life cycle, gene function, transmission, and pathogenesis, as well as developing and testing antiviral therapeutics and vaccines. However, reverse genetics for CoVs has been the most difficult of all positive-sense RNA viruses due to their large genomes, the instability of genomic segments, and the toxicity of some of the viral sequences in bacterial cells (16, 17). It was not until 2000 that the first plasmid (bacterial artificial chromosome, BAC)-launched reverse genetics system of a CoV, transmissible gastroenteritis virus (TGEV), was generated (18). Almazan et al. used natural and engineered restriction sites in the TGEV genome and cloned each fragment, stepwise, into a BAC plasmid (pBeloBAC11), resulting in the assembly of TGEV infectious cDNA clones. In addition, an *in vitro* DNA ligation/T7 RNA polymerase transcription-based approach has been used to generate a reverse genetics system for many CoVs (19–22).

Since the pandemic, several traditional methods, such as *in vitro* DNA ligation/T7 RNA polymerase transcription (19, 23) and restriction sites-based BAC plasmid (24) have been successfully used for SARS-CoV-2 reverse genetics systems. In addition, several new techniques have emerged, including yeast artificial chromosome (YAC) vector/T7 RNA polymerase transcription (25), circular polymerase extension reaction (CPER) (26), infectious subgenomic amplicons (ISA) (27), and cloning-free and exchangeable system for virus engineering and rescue (CLEVER) (28). These reverse genetics systems have played a critical role in understanding SARS-CoV-2 biology, testing antiviral drugs, and designing live-attenuated SARS-CoV-2 vaccine candidates.

In general, plasmids are stable and easy to manipulate, distribute, and store. BACs are excellent plasmids for SARS-CoV-2 reverse genetics systems because they are low-copy number plasmid (one or two copies per cell), stable, tolerant of toxic sequences, and capable of harboring large DNAs. Currently, only one research group has generated and used BAC-based SARS-CoV-2 reverse genetics, relying on natural and engineered restriction sites in the SARS-CoV-2 genome cDNA (24). Similar to the approach used for TGEV reverse genetics (18), they assembled SARS-CoV-2 in a pBeloBAC11 plasmid by multiple rounds of restriction enzyme digestion and ligation. This traditional cloning method is time-consuming, labor-intensive, and often not successful.

To overcome these limitations, we have developed an innovative BAC-based reverse genetics system for SARS-CoV-2. Our approach involves the seamless ligation of the entire SARS-CoV-2 genome in a BAC plasmid within yeast cells using the yeast homologous recombination system. Using this innovative approach, we were able to rapidly generate SARS-CoV-2 WA1, Omicron BA.2.86, and Omicron JN.1 expressing reporters such as mCherry, green fluorescent protein (GFP), and NanoLuc luciferase (Nluc). Using these reporter viruses, we have found that rSARS-CoV-2 BA.2.86 spreads faster and replicates more robustly in primary *ex vivo* cell models, well-differentiated human nasal epithelial (HNE) and human bronchial epithelial (HBE) cultures, compared to rSARS-CoV-2 JN.1 and rSARS-CoV-2 WA1.

## RESULTS

### Rapid assembly of a full-length infectious cDNA clone of SARS-CoV-2 using a yeast-based recombination system

**Fig 1:**
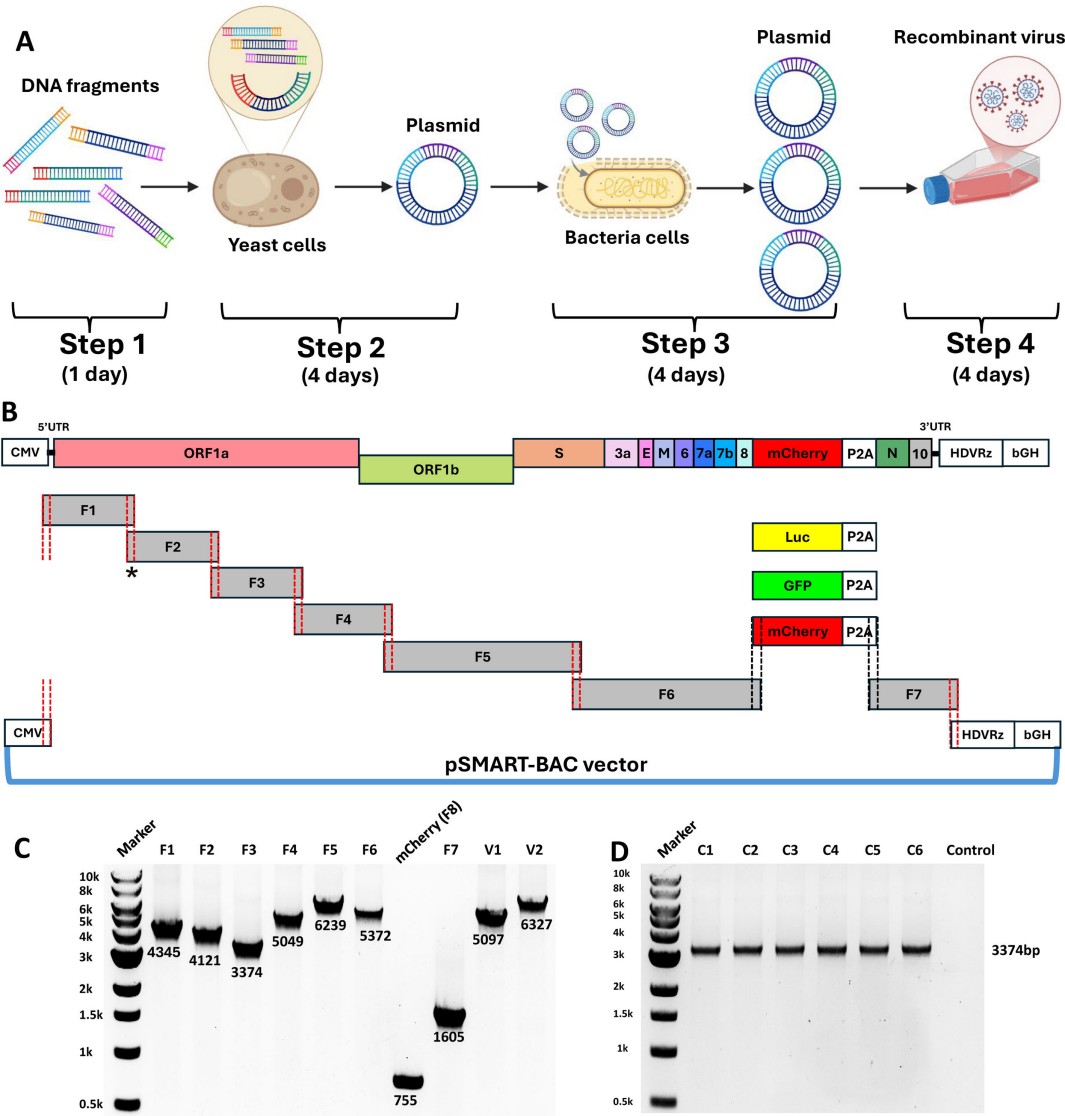
Rapid assembly of a SARS-CoV-2 genome in a BAC plasmid. (**A**) Schematic diagram of the rapid and efficient BAC-based reverse genetics system. The protocol includes four steps. Step 1: DNA fragment preparation. Step 2: recombination in yeast cells. Step 3: large-scale plasmid preparation. Step 4: transfection and virus recovery. The entire procedure only takes less than 2 weeks. (**B**) Schematic diagram of the full-length genomic cDNA clone of SARS-CoV-2 in BAC plasmid. The full-length genomic cDNA clone of SARS-CoV-2 was assembled by ligating seven overlapping fragments (F1 to F7) that cover the entire viral genome into the pSMART-BAC plasmid using a yeast-based homologous recombination system. The pSMART-BAC vector was amplified as two overlapping fragments (V1 and V2). The pSMART-BAC vector was modified to include a yeast replication origin, a URA3 promoter, and a URA3 yeast selection marker, a CMV promoter, a HDVRz, and a bGH termination sequence. After assembly, the full-length SARS-CoV-2 genome is under the control of a CMV promoter at the 5′ end and is flanked at the 3′ end by the HDVRz and bGH termination sequences. A genetic marker, T4303C in the nsp3 gene within the F2 fragment, is marked with a star. This mutation eliminates the TruI site in the nsp3 gene. The reporter gene (F8) mCherry, GFP, or Nluc, flanked by porcine teschovirus-1 2A (P2A), was inserted upstream of the SARS-CoV-2 N gene. (**C**) DNA fragments of SARS-CoV-2 JN.1, mCherry reporter gene, and pSMART vector. The seven overlapping SARS-CoV-2 DNA fragments (F1–F7) covering the entire SARS-CoV-2 JN.1 genome were amplified from viral RNA by RT-PCR. The mCherry reporter gene fragment and two fragments of pSMART vector (V1 and V2) were amplified by PCR. PCR products were resolved in a 1% agarose gel. The size of each DNA fragment is indicated below each band in the gel image. (**D**) Screening positive yeast colonies by PCR. The mixed DNA fragments (from Fig. 1B) were transformed into yeast cells and plated onto SD/Ura⁻ agar plates. After a 2-day incubation at 30°C, individual yeast colonies were picked and cultured overnight in SD/Ura⁻ broth at 30°C. Plasmid was extracted from the yeast culture and used as a template to PCR the fragment 3 (F3, band size: 3,374 bp) of the SARS-CoV-2 genome for initial screening. The agarose gel shows positive yeast colonies for pSMART-SARS-CoV-2 JN.1 mCherry. C1 refers to yeast colony 1. A total of six individual yeast colonies were used for PCR.

We established a yeast-based recombination system for rapid construction of infectious cDNA clones for three SARS-CoV-2 strains: WA1, Omicron BA.2.86, and Omicron JN.1 (Fig. 1A). The pSMART-BAC vector was first modified by insertion of a yeast replication origin, a cytomegalovirus (CMV) promoter, a hepatitis delta virus ribozyme (HDVRz) sequence, and the bovine growth hormone (bGH) transcription termination sequence (Fig. 1B). The SARS-CoV-2 genome was strategically divided into seven overlapping fragments ranging from 1.6 to 6.2 kb in length, with 50 nucleotide (nt) overhangs at their 5′ and 3′ ends. Briefly, viral RNA was extracted from virions of three SARS-CoV-2 strains: WA1; Omicron BA.2.86; or Omicron JN.1. These overlapping fragments (F1–F7) covering the entire SARS-CoV-2 genome were amplified by RT-PCR (Fig. 1C).

To differentiate rSARS-CoV-2 viruses from the parental clinical isolate, a silent mutation (T to C) was introduced in the nsp3 gene (located at the 5′ end of F2) to eliminate the Tru1I restriction site. A DNA fragment (F8) containing the sequences that encode the reporter genes (mCherry, GFP, or Nluc) was designed to insert upstream of the viral nucleocapsid (N) gene (F7) flanked by the porcine teschovirus-1 2A (P2A), which induces ribosomal skipping during translation (29). The pSMART-BAC vector was amplified by PCR as two fragments (V1 and V2) that overlapped with each other at one end and with the terminal fragments of the SARS-CoV-2 genome at the other end (Fig. 1C).

These 10 DNA fragments were mixed and transformed into yeast cells, where they recombined, resulting in an assembly of the complete SARS-CoV-2 genome in the pSMART-BAC plasmid (Fig. 1A). In these BAC plasmids, the SARS-CoV-2 WA1, Omicron BA.2.86, or Omicron JN.1 genome was placed under the control of the CMV promoter, flanked at its 3′ terminus by a 30 nt polyadenylation sequence, followed by the HDVRz and bGH termination sequences (Fig. 1B).

**Fig 2:**
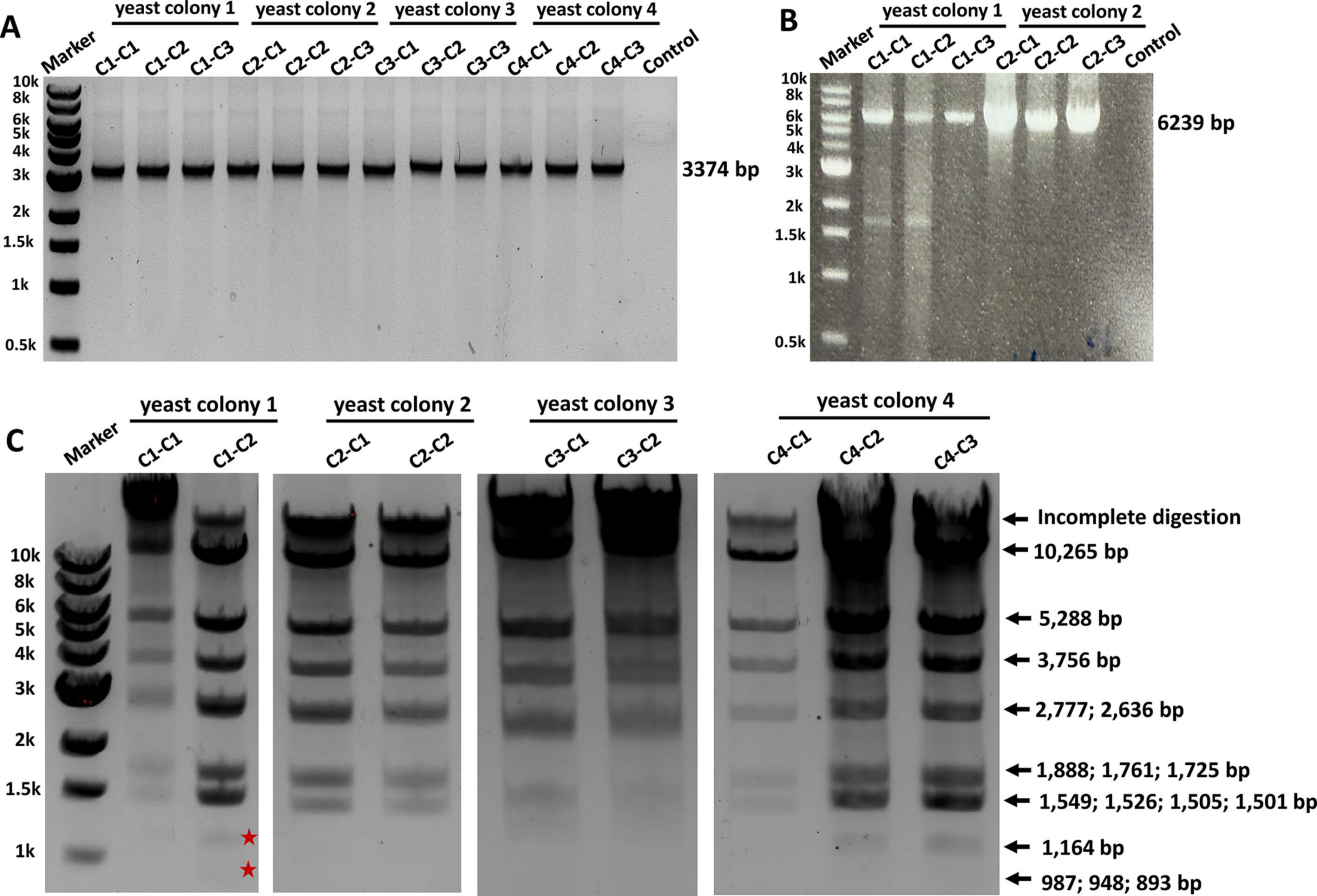
Screening positive bacterial colonies by PCR and restriction enzyme digestion. (**A**) PCR amplification of the F3 fragment from bacteria colonies. The plasmid extracted from yeast cells was transformed into DH10B competent cells by electroporation and grown on LB plates. An individual bacterial colony was picked from the LB plates and grown in 5 mL of LB medium. After 24-hour incubation, 2 µL of bacterial culture was used for PCR screening for SARS-CoV-2 fragment 3 (F3, band size: 3,374 bp). The agarose gel shows positive bacterial colonies for pSMART-SARS-CoV-2 JN.1 mCherry. C1-1 refers to the bacterial colony 1 from yeast colony 1. A total of four yeast colonies and three bacterial colonies from each yeast colony were used for PCR screening. (**B**) PCR amplification of F5 fragment from bacterial colonies. Two microliters of bacterial culture was used for PCR screening for SARS-CoV-2 fragment 5 (F5, band size: 6,239 bp). Agarose gel shows positive bacterial colonies for pSMART-SARS-CoV-2 JN.1 mCherry. C1-1 refers to bacterial colony 1 from yeast colony 1. Two yeast colonies and three bacterial colonies from each yeast colony were used for PCR screening. (**C**) Restriction enzyme digestion of plasmid extracted bacteria. If PCR screening is positive, plasmids were extracted from two bacterial colonies for each yeast colony. The plasmid was digested by HindIII and analyzed in a 1% agarose gel. The red stars in the left panel indicate two visible weak bands.

The BAC plasmid was extracted from yeast colonies and initially screened by PCR using two primers annealing to the F3 fragment. All six yeast colonies were positive by PCR (Fig. 1D). Plasmids from yeast colonies 1–4 were transformed into *E. coli* DH10B competent cells to generate a bulk stock of the plasmid. For each transformed yeast plasmid, three bacterial colonies were picked and screened by PCR for F3. All were positive (Fig. 2A). The three bacterial colonies from yeast colonies 1 and 2 were used as templates for PCR for F5. All were positive (Fig. 2B). Two to three bacterial plasmids from each yeast colony were further screened by digestion with HindIII, which generates 16 DNA bands. Some of these bands had similar sizes and could not be resolved in agarose gel (Fig. 2C). All these plasmids generated multiple fragments which are consistent with the predicted HindIII digestion pattern (Fig. 2C), suggesting that the complete SARS-CoV-2 genome had been inserted into the pSMART-BAC plasmid.

Finally, the plasmids were deep sequenced. All plasmids contained the correct sequence of the SARS-CoV-2 genome and the genetic marker for the recombinant genome: the lack of the Tru1I site from the *nsp3* gene (F2). No additional mutations were found anywhere in the genome. Using this method, we rapidly constructed a total of nine plasmids: pSMART-BAC containing SARS-CoV-2 WA1 genome with inserted reporters (pSMART-WA1-GFP, pSMART-WA1-mCherry, and pSMART-WA1-Nluc), pSMART-BAC containing SARS-CoV-2 Omicron BA.2.86 genome with inserted reporters (pSMART- BA.2.86-GFP, pSMART-BA.2.86-mCherry, and pSMART-BA.2.86-Nluc), and pSMART-BAC containing SARS-CoV-2 Omicron JN.1 genome with inserted reporters (pSMART-JN.1-GFP, pSMART-JN.1-mCherry, and pSMART-JN.1-Nluc). The entire protocol is easy, rapid, and highly efficient. Most yeast clones and bacterial clones are correct. It takes less than 10 days to generate a BAC plasmid harboring the full-length SARS-CoV-2 genomic cDNA (Fig. 1A).

### Rescue of rSARS-CoV-2 expressing reporters

**Fig 3:**
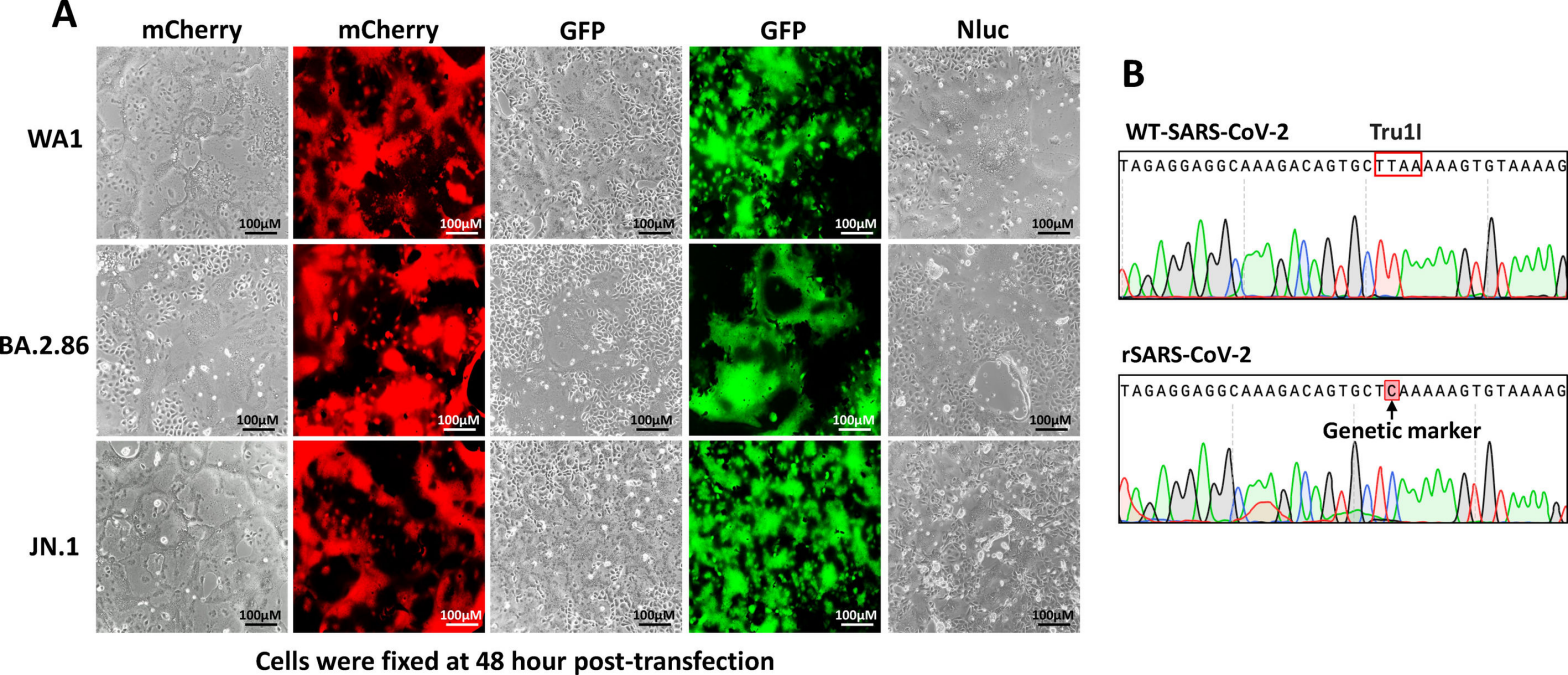
Recovery of rSARS-CoV-2 WA1, BA.2.86, and JN.1 expressing mCherry, GFP, and Nluc. (**A**) SARS-CoV-2-induced cytopathic effects (CPE) after transfection of plasmids in Vero E6 TMPRSS2-T2A-ACE2 cells. Vero E6-TMPRSS2-T2A-ACE2 cells in a 12-well plate were transfected with 2 µg of pSMART-BAC-SARS-CoV-2 plasmid encoding SARS-CoV-2 genome with a reporter gene. CPE was observed at day 2 post-transfection for each plasmid. Robust fluorescent signals were observed under fluorescent microscopy at day 2 for rSARS-CoV-2 mCherry and GFP viruses, confirming the successful rescue of recombinant viruses. (**B**) Sanger sequencing of the *nsp3* gene of the WT SARS-CoV-2 and the rSARS-CoV-2 showed that all recombinant viruses contain a silent mutation (T4303C) genetic marker (representative figure).

To recover recombinant SARS-CoV-2 (rSARS-CoV-2), Vero E6-TMPRSS2-T2A-ACE2 cells in 12-well plates were transfected with 2 μg of pSMART-BAC encoding a SARS-CoV-2 genome. Cytopathic effects (CPE) and fluorescent signals were monitored daily. By 48 h post-transfection, all nine rSARS-CoV-2 were successfully rescued. Extensive cell-cell fusion, resulting in syncytia, was observed, a hallmark of SARS-CoV-2-induced CPE (Fig. 3A). GFP or mCherry fluorescent signals were detected in cells transfected with pSMART-BAC encoding the SARS-CoV-2 genome containing the GFP or mCherry insertion, confirming the robust expression of the reporter genes by these recombinant viruses. At day 3 post-transfection, cell culture supernatants were harvested and used for inoculating fresh Vero E6-TMPRSS2-T2A-ACE2 cells in T25 flasks. At day 2 post-infection, all nine recombinant viruses reached maximal CPE. Cell culture supernatants were harvested, aliquoted, and stored at −80°C for further characterization. These recombinant viruses were named rWA1-GFP, rWA1-mCherry, rWA1-Nluc, rBA.2.86-GFP, rBA.2.86-mCherry, rBA.2.86-Nluc, rJN.1-GFP, rJN.1-mCherry, and rJN.1-Nluc. Subsequently, 100 μL of virus stock was treated with 2 μL of DNase to eliminate any plasmid DNA contamination, and total RNA was extracted using Trizol. The eight fragments covering the entire SARS-CoV-2 genome were amplified by RT-PCR and sent for Sanger sequencing. We confirmed the presence of the genetic mark (T to C mutation in the nsp3 gene) in all recombinant viruses, while it was absent in the parental clinical isolates (Fig. 3B). All recombinant viruses contain the targeted reporter gene. No additional mutations were found in any of these recombinant viruses.

**Fig 4:**
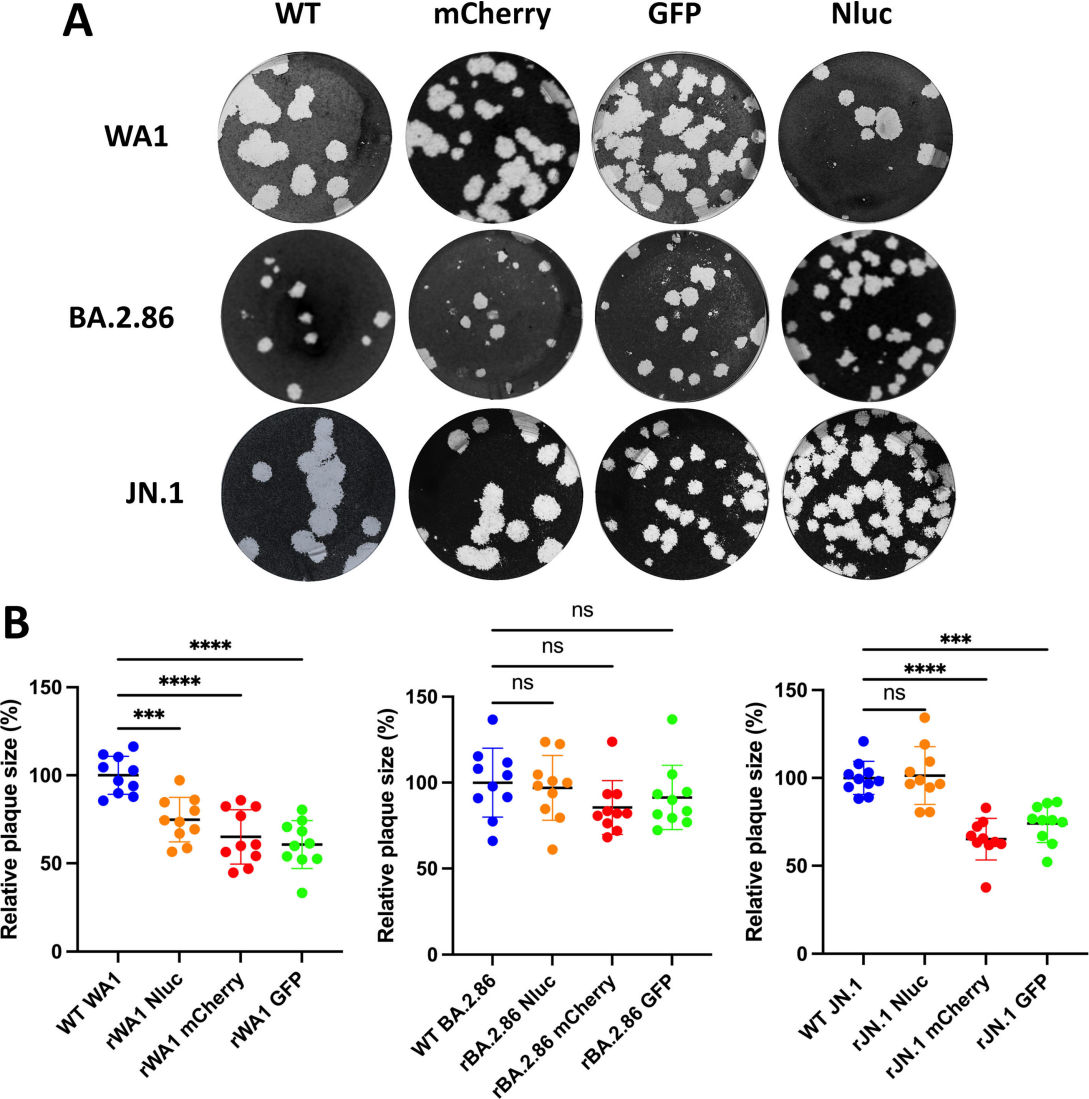
Plaque morphology of recombinant viruses. (**A**) Plaque morphology. A plaque assay was conducted for each recombinant virus in Vero E6 TMPRSS2-T2A-ACE2 cells in 12-well plates. After incubation at 37°C for 2 days, cells were fixed with 4% paraformaldehyde for 1 h. Viral plaques were visualized by staining with 0.05% (vol/vol) crystal violet. (**B**) Plaque size. The diameter of 10 plaques from each recombinant virus was measured by Image J. The average diameter of 10 plaques was shown. The plaque size of reporter viruses was compared to their parental virus. Data were analyzed using one-way ANOVA (ns indicates not significant; ***P < 0.001; ****P < 0.0001). The *P* values in the right panel (from left to right) were 0.9902, <0.0001, and 0.0001; in the middle panel were 0.9651, 0.2050, and 0.5910; and in the left panel were 0.0004, <0.0001, and <0.0001.

Both rWA1 and rJN.1 formed large plaques in Vero E6-TMPRSS2-T2A-ACE2 cells, whereas rBA.2.86 produced smaller plaques (Fig. 4A). The insertion of the reporter genes reduced the plaque sizes in the WA1 and JN.1 backbones, but not noticeably in the BA.2.86 backbone (Fig. 4B).

### Insertion of a reporter gene into SARS-CoV-2 does not significantly alter its replication

**Fig 5:**
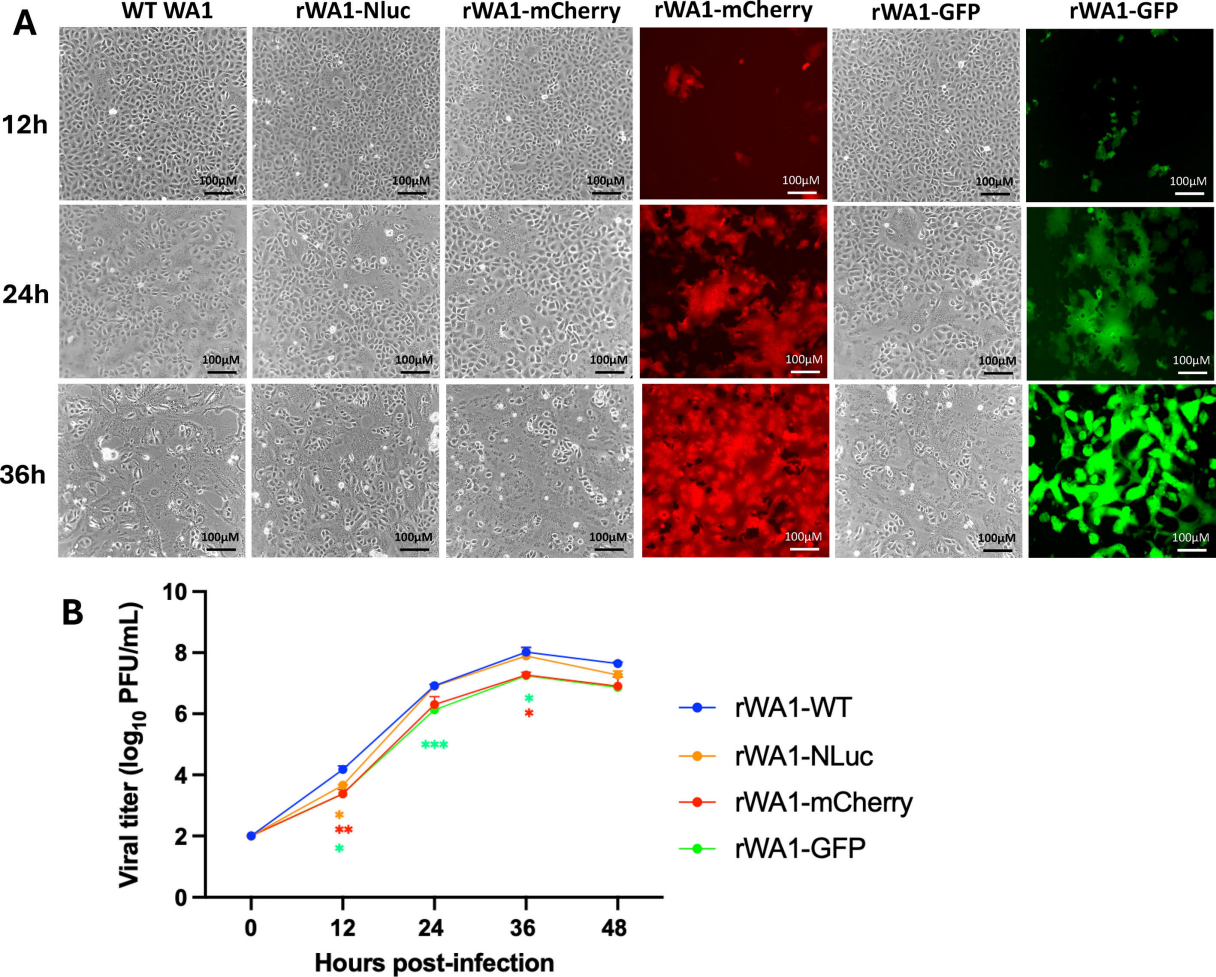
Replication kinetics of rSARS-CoV-2 WA1 expressing mCherry, GFP, or Nluc. (**A**) Progression of CPE and fluorescent signal. Vero E6-TMPRSS2-T2A-ACE2 cells were infected with each recombinant virus at an MOI of 0.01. At 12, 24, and 36 h post-infection, representative images of CPE and fluorescent signal (mCherry or GFP) from virus-infected cells were captured by microscopy. The CPE and fluorescent images were from different fields of view. (**B**) Replication kinetics of recombinant viruses. Supernatants were collected from above infection (*n* = 3) at 0, 12, 24, 36, and 48 h post-infection, and viral titer was determined by plaque assay. Data were analyzed using one-way ANOVA (*P < 0.05; **P < 0.01; ***P < 0.001). Data on each time was compared to those of rWA-WT. The *P* values at 12 h (from top to bottom) were 0.0388, 0.006, and 0.0192; at 24 h was 0.0002; and at 36 h were 0.0322 and 0.0154.

**Fig 6:**
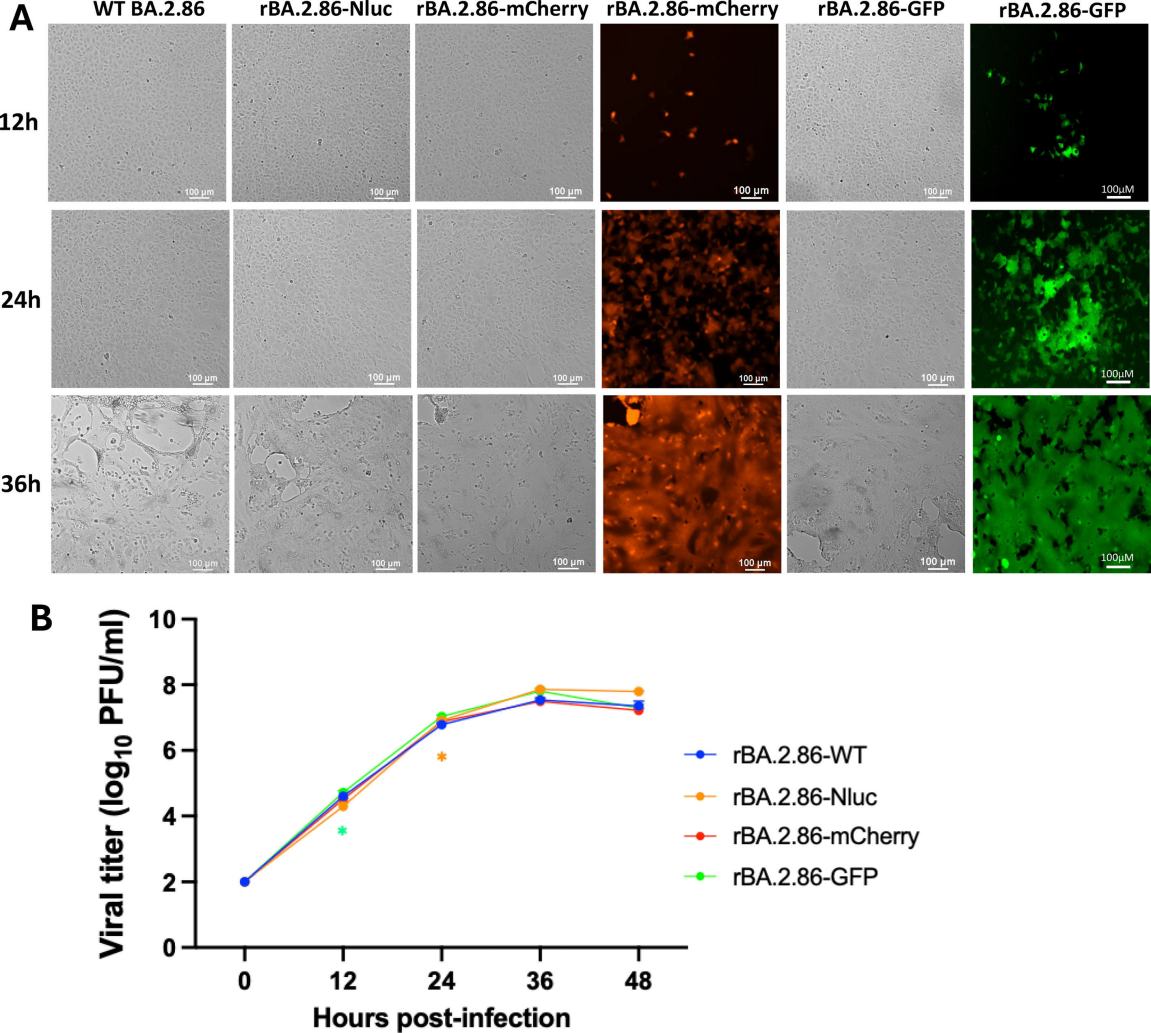
Replication kinetics of rBA.2.86 expressing mCherry, GFP, or Nluc. (**A**) Progression of CPE and fluorescent signal. Vero E6-TMPRSS2-T2A-ACE2 cells were infected with each recombinant virus at an MOI of 0.01. At 12, 24, and 36 h post-infection, representative images of CPE and fluorescent signal (mCherry or GFP) from virus-infected cells were captured by microscopy. The CPE and fluorescent images were from different fields of view. (**B**) Replication kinetics of recombinant viruses. Supernatants were collected from above infection (*n* = 3) at 0, 12, 24, 36, and 48 h post-infection, and viral titer was determined by plaque assay. Data were analyzed using one-way ANOVA (*P < 0.05). Data on each time was compared to those of rWA-WT. The *P* values shown at 12 h and 24 h were 0.0275 and 0.0118, respectively.

**Fig 7:**
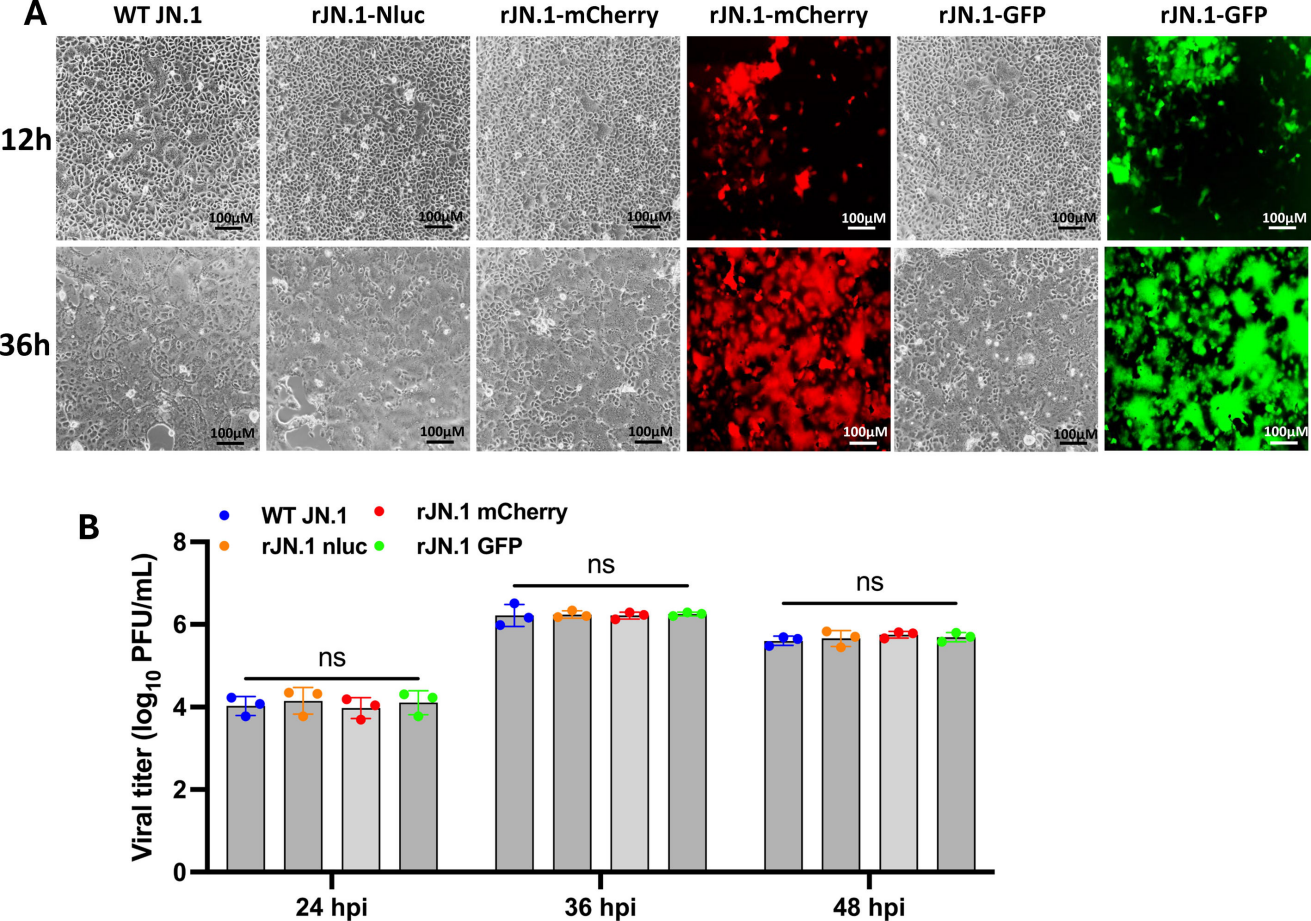
Replication kinetics of rJN.1 expressing mCherry, GFP, or Nluc. (**A**) Progression of CPE and fluorescent signal. Vero E6-TMPRSS2-T2A-ACE2 cells were infected with each recombinant virus at an MOI of 0.01. At 12 and 36 h post-infection, representative images of CPE and fluorescent signal (mCherry or GFP) from virus-infected cells were captured by microscopy. The CPE and fluorescent images were from different fields of view. (**B**) Replication kinetics of recombinant viruses. Supernatants were collected from above infection (*n* = 3) at 24, 36, and 48 h post-infection, and viral titer was determined by plaque assay. Data were analyzed using one-way ANOVA (ns, not significant).

Next, we determined whether the insertion of reporter genes into SARS-CoV-2 affects viral replication. Briefly, confluent Vero E6-TMPRSS2-T2A-ACE2 cells were inoculated with WT WA1, rWA1-GFP, rWA1-mCherry, or rWA1-Nluc at an MOI of 0.01, and CPE and fluorescent signals were monitored (Fig. 5A). All recombinant viruses had developed small syncytia at 12 h post-infection and exhibited extensive syncytia at 36 h. Strong GFP and mCherry signals were observed at 36 h in rWA1-GFP- and rWA1-mCherry–infected cells, respectively. By 48 h, most cells in the culture had died. Cell culture supernatants were collected at 0, 12, 24, 36, and 48 h for virus titration. All viruses reached their peak titers by 36 h. rWA1-GFP and rWA1-mCherry displayed slightly reduced virus replication, whereas the replication kinetics of rWA1-Nluc and WT-WA1 were higher and indistinguishable (Fig. 5B). Next, Vero E6-TMPRSS2-T2A-ACE2 cells were inoculated with WT BA.2.86, rBA.2.86-GFP, rBA.2.86-mCherry, or rBA.2.86-Nluc. All these recombinant viruses had a similar pattern of CPE progression (Fig. 6A) and replicated similarly (Fig. 6B) in Vero E6-TMPRSS2-T2A-ACE2 cells. Finally, Vero E6-TMPRSS2-T2A-ACE2 cells were inoculated with WT JN.1, rJN.1-GFP, rJN.1-mCherry, or rJN.1-Nluc. No significant difference in replication kinetics (*P* > 0.05) or progression of CPE was observed among these recombinant viruses (Fig. 7). Therefore, these results demonstrate that the insertion of the reporter gene into SARS-CoV-2 WA1, Omicron BA.2.86, and JN.1 does not significantly affect SARS-CoV-2 replication.

### Omicron BA.2.86 spreads much faster than JN.1 in both HNE and HBE cultures

**Fig 8:**
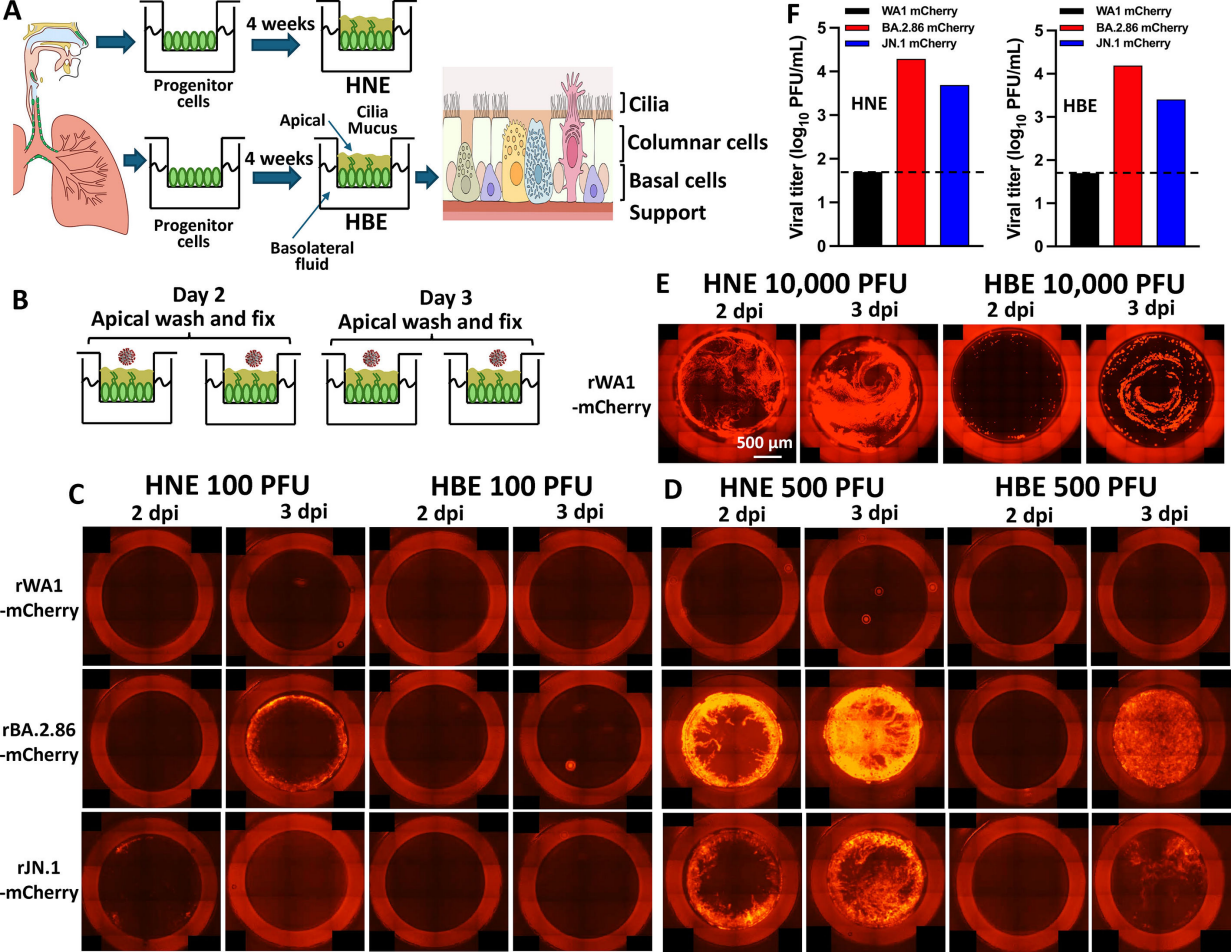
Spread of WA1-mCherry, BA.2.86-mCherry, and JN.1-mCherry in HNE and HBE cultures at 100 and 500 PFU. (**A**) Diagram of producing HNE and HBE cultures. Human nasal or lung progenitor cells derived from donors were seeded on collagen-coated 6.5 mm transwells and were differentiated at the air-liquid interface in the presence of puromycin (2 μg/mL) to generate well-differentiated, polarized HNE and HBE cultures. (**B**) Experimental design. HNE (*n* = 2) and HBE (*n* = 2) cultures from donor 1 grown in 24-well transwell plates were inoculated with 100 (**C**) or 500 (**D**) PFU of rWA1-mCherry, rBA.2.86-mCherry, or rJN.1-mCherry, or 10,000 PFU (**E**) of rWA1-mCherry. At days 2 and 3, two transwells of HNE (*n* = 2) and HBE (*n* = 2) cultures were fixed in 10% formalin, viral spread was monitored by fluorescence microscopy, and the entire transwell was scanned by Image J. Representative micrographs at each time point are shown. (**C**) Virus spread in HNE and HBE cultures at 100 PFU. (**D**) Virus spread in HNE and HBE cultures at 500 PFU. (**E**) Virus spread in HNE and HBE cultures at 10,000 PFU. (**F**) Viral titer released from apical surface. At day 3, apical washes were collected from each transwell for virus titration by plaque assay. Viral titer was the average from two transwells (*n* = 2). Dotted line indicates the detection limit. Scale bar for all transwell images is 500 μm.

The primary targets of SARS-CoV-2 are the upper and lower respiratory tract airway epithelia. Thus, well-differentiated primary human nasal epithelial (HNE) cultures and human bronchial epithelial (HBE) cultures are excellent “near *in vivo*” human epithelial models for SARS-CoV-2 infection (30). Human nasal epithelial cells are collected by nasal brushing of volunteer donors, and basal cells are isolated and differentiated into HNE cultures at the air-liquid interface (ALI), mimicking the human nasal mucosa (31, 32). Bronchial epithelial basal cells are isolated from the bronchi of organ donors and differentiated at the ALI, mimicking the environment of the human lung bronchus (Fig. 8A). After 4 weeks of differentiation at the ALI, HNE and HBE cultures are pseudostratified, containing columnar goblet cells that produce mucus, columnar ciliated cells whose cilia beat to move the mucus around the well, and several other minor cell types. The tight junctions near the top of these columnar cells maintain their polarization and are a barrier to pathogens (33–35).

To compare the dynamics of replication and spread of the SARS-CoV-2 WA1, BA.2.86, and JN.1 viruses, we used their mCherry-expressing versions to inoculate HNE and HBE cultures from donor 1 (Fig. 8B). We used two low virus doses, 100 PFU and 500 PFU, of each virus. These titers were determined in Vero E6-TMPRSS2-T2A-ACE2 cells, and are equivalent to MOIs of 1.7 × 10^−4^ and 8.3 × 10^−4^, respectively. Briefly, the apical surface of HNE (*n* = 4) and HBE (*n* = 4) cultures in transwells was washed three times with DMEM to remove the mucus, then inoculated with 100 μL of DMEM containing 100 or 500 PFU of rBA.2.86-mCherry, rJN.1-mCherry, or rWA1-mCherry and incubated at 37°C. To ensure that we would be able to detect infection of both HBE and HNE cultures, we also inoculated four additional HNE (*n* = 4) and HBE (*n* = 4) transwells with 10,000 PFU of rWA1-mCherry in 100 μL of DMEM, corresponding to an MOI of 1.7 × 10^−2^. After a 2-hour inoculation, the inoculum was removed, the apical surface was washed with 100 μL of DMEM, and the transwells were incubated at 37°C for 3 days.

On days 2 and 3 post-inoculation, the apical surface of all the inoculated HNE and HBE cultures was washed with 100 μL of DMEM, and the virus produced was titrated. After washing, the transwells were fixed in 10% formalin, and the entire transwell was scanned using the Image J software. At 100 PFU of infection, no mCherry signal was observed in rWA1-mCherry-inoculated or rJN.1-mCherry-inoculated HNE or HBE cultures (Fig. 8C). However, the mCherry signal was visible in HNE cultures inoculated with rBA.2.86-mCherry at day 3 (Fig. 8C), but no obvious mCherry expression was observed in the HBE cultures (Fig. 8C). When the inoculum dose in HNE culture was increased to 500 PFU, rBA.2.86-mCherry produced mCherry on day 2, which spread to the entire well by day 3 (Fig. 8D). rJN.1-mCherry also displayed mCherry expression at days 2 and 3 in the HNE culture, but the signal was much weaker than the rBA.2.86-mCherry infection (Fig. 8D). When HBE cultures were inoculated with 500 PFU of each virus, rBA.2.86-mCherry spread faster than rJN.1-mCherry (Fig. 8D). By day 3, the entire transwell was red in the rBA.2.86-mCherry–infected HBE cultures. rJN.1-mCherry produced no mCherry expression at day 2, but mCherry expression was observed by day 3 (Fig. 8D). In contrast, no mCherry was detected in HNE or HBE cultures inoculated with 500 PFU of rWA1-mCherry. However, when 10,000 PFU of rWA1-mCherry was used, mCherry expression was observed in both HNE and HBE cultures at day 2, and virus spread had increased by day 3 (Fig. 8E). Apical washes from HNE and HBE cultures at day 3 were used for virus titration by plaque assay. Viral titers from rBA.2.86-mCherry–infected HNE (*n* = 2) and HBE (*n* = 2) cultures were higher than those from rJN.1-mCherry–infected cultures (Fig. 8F). Together, these data indicate that the spread of rBA.2.86-mCherry and rJN.1-mCherry in HNE and HBE cultures significantly increased compared to the rWA1-mCherry and that the spread of rBA.2.86-mCherry was faster than rJN.1-mCherry in both HNE and HBE cultures.

**Fig 9:**
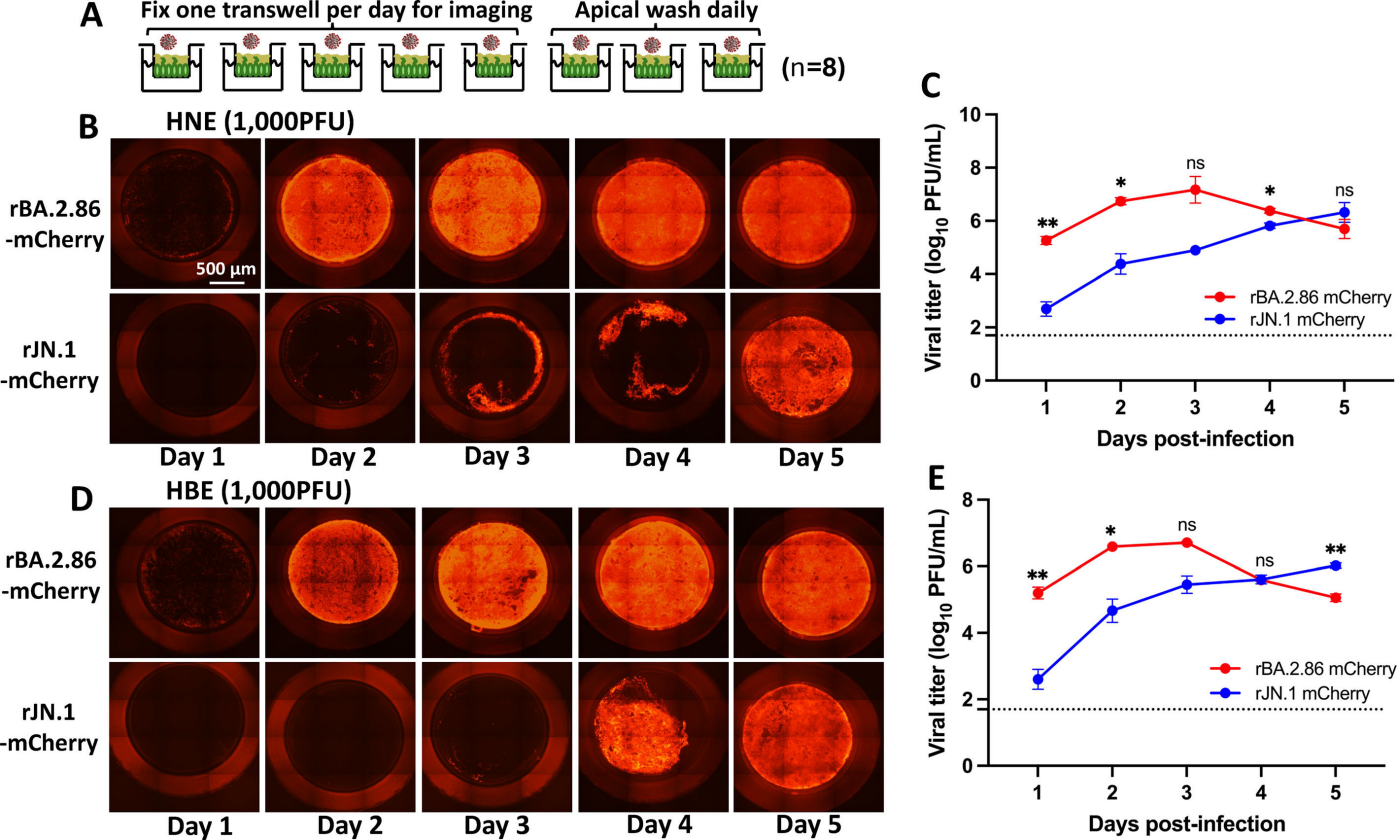
Replication and spread of BA.2.86-mCherry and JN.1-mCherry in HNE and HBE cultures at 1,000 PFU. (**A**) Diagram of experimental design. HNE (*n* = 8) and HBE (*n* = 8) cultures from donor 2 were grown in 24-well transwell plates and were inoculated with 1,000 PFU of rBA.2.86-mCherry or rJN.1-mCherry. After inoculation, five transwells of HNE (*n* = 5) and HBE (*n* = 5) cultures were used for monitoring virus spread for 5 days, and the other three transwells of HNE (*n* = 3) and HBE (*n* = 3) cultures were used for apical washes and virus titration. (**B**) Virus spread in HNE culture at 1,000 PFU. At days 1–5, the HNE culture was fixed in 10% formalin (one transwell per day), viral spread was visualized by a fluorescence microscopy, and scanned by Image J. (**C**) Virus replication in HNE culture at 1,000 PFU. Apical washes from three transwells of HNE (*n* = 3) cultures were collected at 1, 2, 3, 4, and 5 dpi. Viral titers were determined by plaque assay in Vero E6-TMPRSS2-T2A-ACE2 cells. The *P* values from left to right were 0.0031, 0.0225, 0.0618, 0.0115, and 0.4228. (**D**) Virus spread in HBE culture at 1,000 PFU. At days 1–5, the HBE culture was fixed in 10% formalin (one transwell per day), viral spread was visualized by a fluorescence microscopy, and scanned by Image J. Scale bar for all transwell images is 500 μm. (**E**) Virus replication in HBE culture at 1,000 PFU. Apical washes from three transwells of HBE (*n* = 3) cultures were collected at 1, 2, 3, 4, and 5 dpi. Viral titers were determined by plaque assay in Vero E6-TMPRSS2-T2A-ACE2 cells. Viral titers were the average of 3 transwells ± standard deviation. The *P* values from left to right were 0.0034, 0.0322, 0.0664, >0.9999, and 0.0022. Data were analyzed using two-way ANOVA (ns, not significant; *P < 0.05; **P < 0.01).

We next conducted a second infection experiment using HNE and HBE cultures derived from a different donor (donor 2). The HNE (*n* = 8) and HBE (*n* = 8) cultures were inoculated with 1,000 PFU (equivalent to MOI of 1.7 × 10^−3^) of rBA.2.86-mCherry or rJN.1-mCherry (Fig. 9A). Among these eight transwells, one was fixed with formalin each day for 5 days and scanned using Image J. The other three transwells were used for apical washes to determine viral titers (Fig. 9A). rBA.2.86-mCherry replicated and spread rapidly, infecting the entire transwell by 2 days post-infection (dpi), reaching peak titer (10^7^ PFU/mL) at 3 dpi, and declining at 4 and 5 dpi in both HNE (Fig. 9B and C) and HBE cultures (Fig. 9D and E). In comparison, the spread and growth kinetics of rJN.1-mCherry were much slower than rBA.2.86-mCherry (Fig. 9B through E). Viral titers on 1–3 dpi in rJN.1-mCherry were significantly lower than rBA.2.86-mCherry. By 5 dpi, rJN.1-mCherry had infected the entire well and reached a peak titer of 10^6^ PFU/mL.

**Fig 10:**
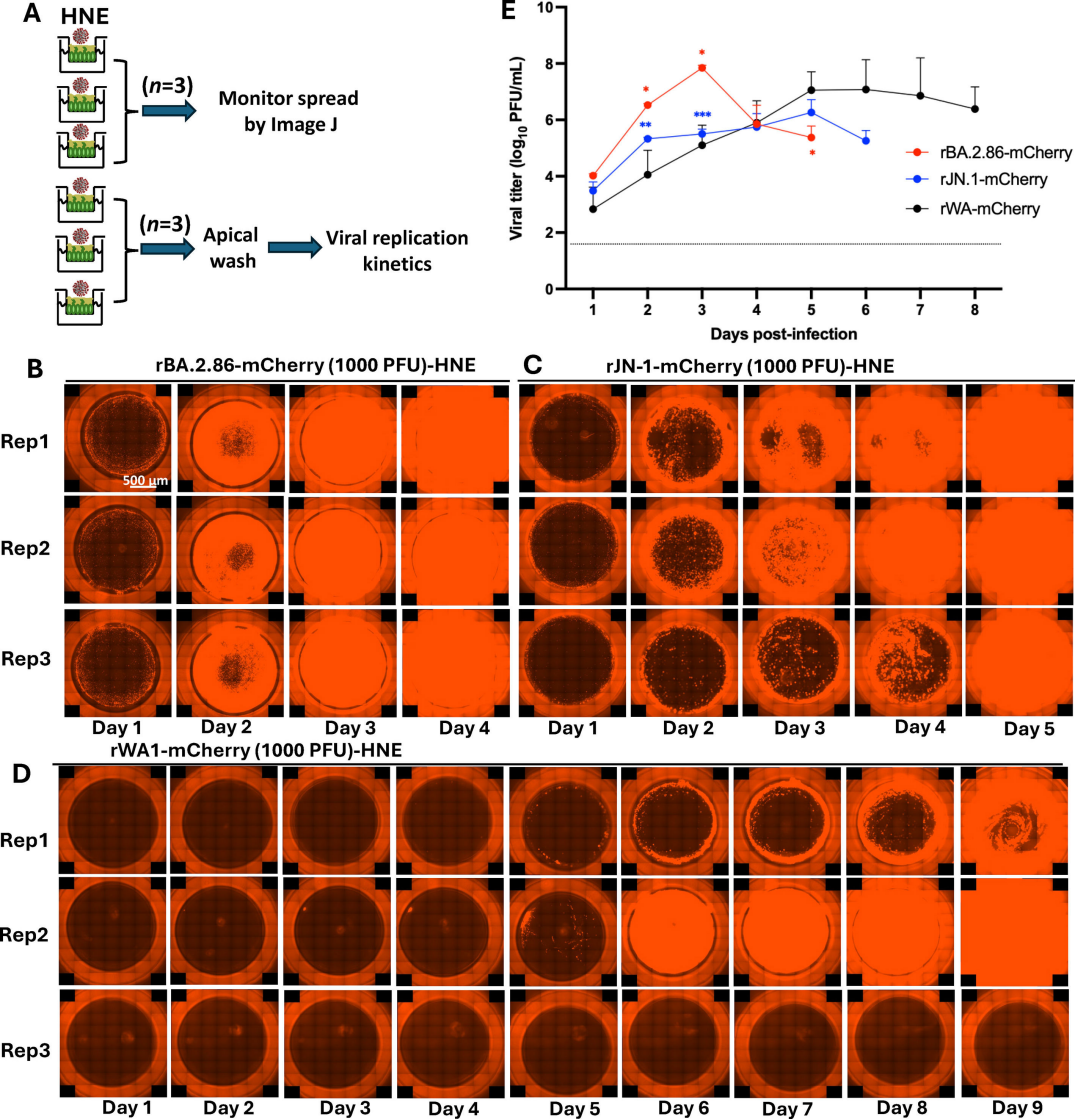
Spread and replication of rWA1-mCherry, rBA.2.86-mCherry, and rJN.1-mCherry in HNE cultures at 1,000 PFU. (**A**) Experimental design. HNE (*n* = 6) cultures from donor 3 were grown in 24-well transwell plates and inoculated with 1,000 PFU of rBA.2.86-mCherry (**B**), rJN.1-mCherry (**C**), or rWA1-mCherry (**D**). Three HNE (*n* = 3) cultures were used for monitoring the spread of mCherry signal daily by fluorescence microscopy until the mCherry spread to the entire transwell, and scanned by Image J. The scale bar for all transwell images is 500 μm. (**E**) Virus replication kinetics in HNE culture. Apical washes were collected daily from the other three HNE (*n* = 3) cultures infected by each recombinant virus. Viral titer in apical washes was determined by plaque assay in Vero E6-TMPRSS2-T2A-ACE2 cells. Viral titers were the average of 3 transwells ± standard deviation. Data were analyzed using two-way ANOVA and one-way ANOVA (*P < 0.05; **P < 0.01; ***P < 0.001). The red stars indicate the statistical differences of rBA.2.86-mCherry compared to the rWA1-mCherry (the *P* values shown were 0.0383, 0.0199, and 0.0267). The blue stars indicate the statistical differences of rJN1-mCherry compared to the rBA.2.86-mCherry (the *P* values shown were 0.0043 and 0.0002).

**Fig 11:**
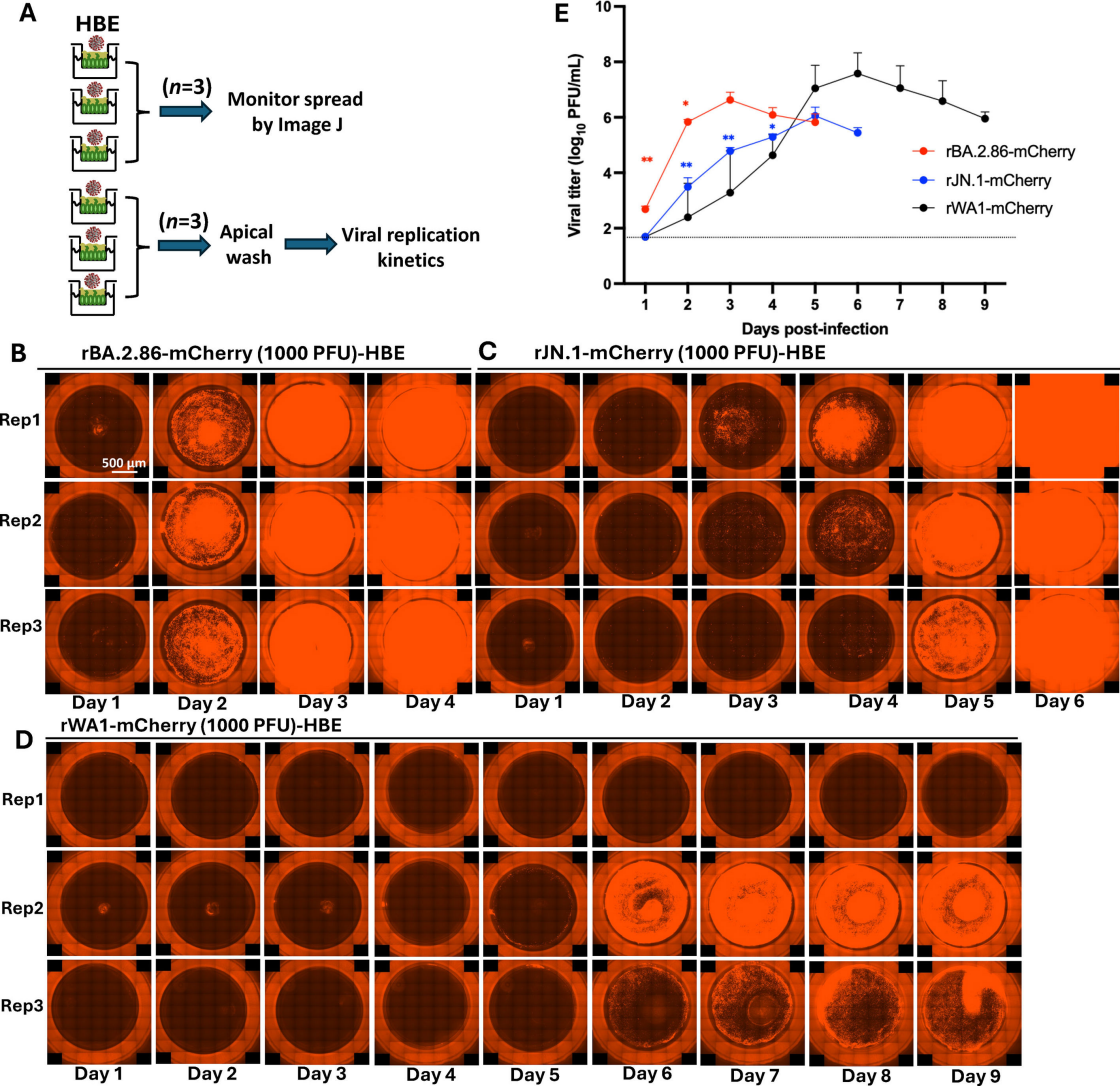
Spread and replication of rWA1-mCherry, rBA.2.86-mCherry, and rJN.1-mCherry in HBE cultures at 1,000 PFU. (**A**) Experimental design. HBE (*n* = 6) cultures from donor 3 were grown in 24-well transwell plates and were inoculated with 1,000 PFU of rBA.2.86-mCherry (**B**), rJN.1-mCherry (**C**), or rWA1-mCherry (**D**). Three HBE (*n* = 3) cultures were used for monitoring the spread of mCherry signal daily by fluorescence microscopy until the mCherry spread to the entire transwell and scanned by Image J. Scale bar for all transwell images is 500 μm. (**E**) Virus replication kinetics in HBE culture. Apical washes were collected daily from the other three HBE (*n* = 3) cultures infected by each recombinant virus. Viral titer in apical washes was determined by plaque assay in Vero E6-TMPRSS2-T2A-ACE2 cells. Viral titers were the average of 3 transwells ± standard deviation. Data were analyzed using two-way ANOVA and one-way ANOVA (*P < 0.05; **P < 0.01). The red stars indicate the statistical differences of rBA.2.86-mCherry compared to the rWA1-mCherry (the *P* value shown was 0.004 and 0.0406). The blue stars indicate the statistical differences of rJN1-mCherry compared to the rBA.2.86-mCherry (the *P* values shown were 0.0041, 0.0024, and 0.0204).

The finding that rBA.2.86-mCherry spreads and replicates faster than rJN.1-mCherry is somewhat surprising because Omicron JN.1, but not the Omicron BA.2.86 variant, became dominant worldwide from late 2023 to May 2024. To further confirm this finding, we conducted a more comprehensive infection experiment using HNE and HBE cultures derived from a third donor (donor 3). Specifically, we determined the dynamics of virus spread and replication in HNE (*n* = 6) and HBE (*n* = 6) cultures using 1,000 PFU (MOI of 1.7 × 10^−3^) or 200 PFU (MOI of 3.3 × 10^−4^) of each virus (Fig. 10A). Among these six transwells, three transwells were used for monitoring virus spread until the mCherry signal spread throughout the entire transwell, and the apical surface of the other three transwells was washed daily for determination of viral titer. At 1,000 PFU, rBA.2.86-mCherry had the fastest spread and virus production in HNE cultures (Fig. 10B). The mCherry signal included nearly all the transwells as early as day 2 post-infection. rJN.1-mCherry was the second fastest virus: two out of three transwells were fully covered by mCherry signal by day 4, and the third transwell turned red by day 5 (Fig. 10C). rWA1-mCherry was the slowest virus: two out of three transwells had weak and sporadic mCherry signal on day 5, and mCherry signal only spread to the entire transwell by days 6 and 9 (Fig. 10D). The third well had no or little virus spread even at day 9 (Fig. 10D). Next, viral titer released from the apical surface of HNE cultures in the other three transwells was determined by plaque assay. rBA.2.86-mCherry and rJN.1-mCherry reached peak titer on days 3 and 5, respectively (Fig. 10E). However, replication of rWA1-mCherry was significantly delayed compared to the rBA.2.86-mCherry and rJN.1-mCherry viruses (Fig. 10E). Similar results were observed in HBE cultures (Fig. 11). rBA.2.86-mCherry spread faster than rJN.1-mCherry (Fig. 11). Robust mCherry signal was observed at day 2, and the entire transwell had turned red by day 3 in rBA.2.86-mCherry–infected HBE culture (Fig. 11B), whereas rJN.1-mCherry spread to the entire transwell by day 5 or 6 (Fig. 11C). rWA1-mCherry had the slowest spread in HBE cultures. Only one out of three transwells had robust mCherry expression with mCherry reaching the entire transwell by day 6; the second transwell had moderate mCherry expression from days 6 to 9 but did not reach the entire transwell at day 9; and the third transwell had no or little mCherry expression even by day 9 (Fig. 11D). Apical washes were collected from HBE culture for virus titration by plaque assay. rBA.2.86-mCherry and rJN.1-mCherry reached peak viral titers at days 3 and 5, respectively (Fig. 11E). rWA1-mCherry had a significant delay in replication from days 1 to 4 and reached peak titer by day 6 (Fig. 11E).

**Fig 12:**
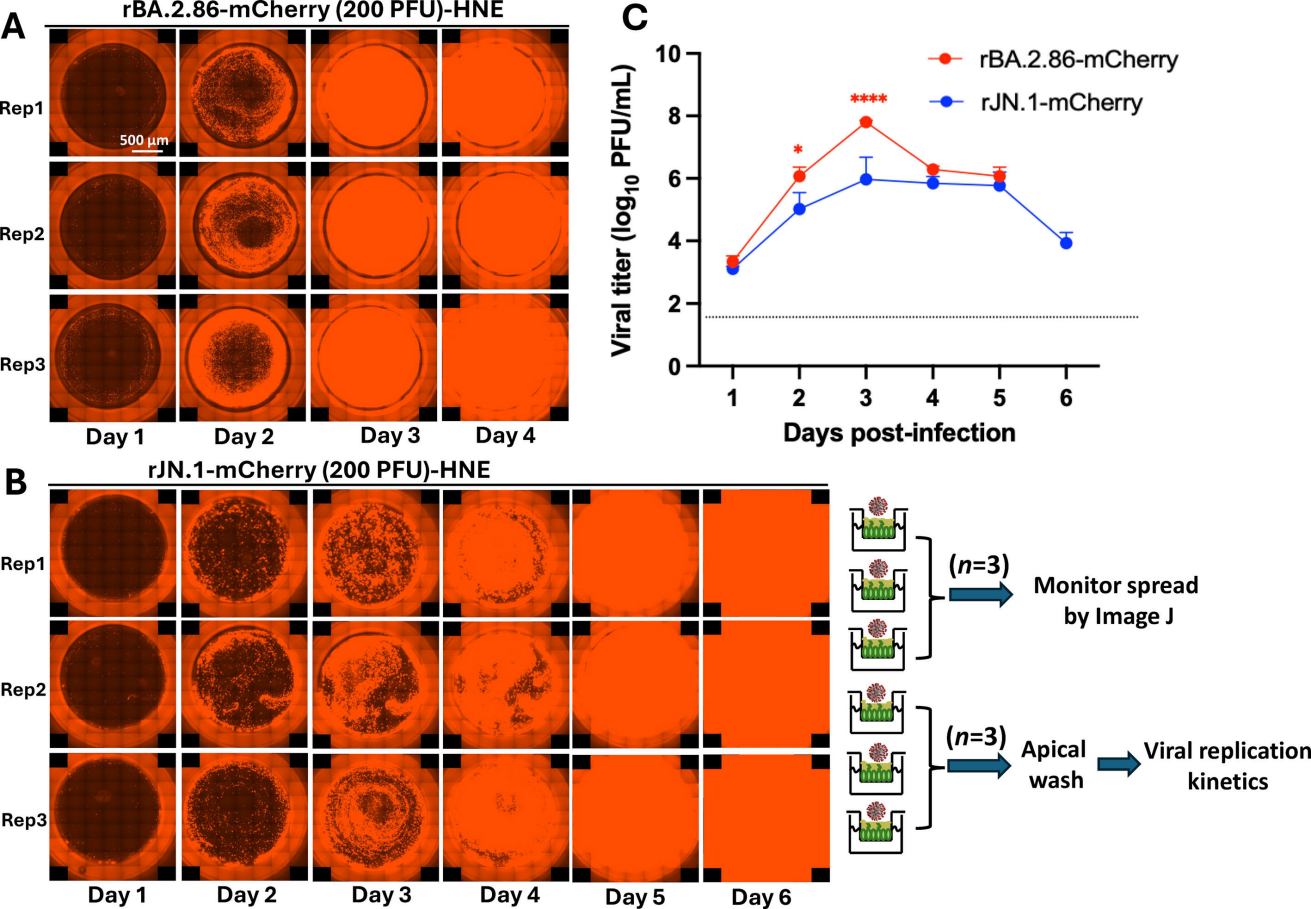
Spread and replication of rBA.2.86-mCherry and rJN.1-mCherry in HNE cultures at 200 PFU. HNE (*n* = 6) cultures from donor 3 were grown in 24-well transwell plates and were inoculated with 200 PFU of rBA.2.86-mCherry (**A**) or rJN.1-mCherry (**B**). Three HNE (*n* = 3) cultures were used for monitoring the spread of mCherry signal daily by fluorescence microscopy until the mCherry spread to the entire transwell and was scanned by Image J. Scale bar for all transwell images is 500 μm. (**C**) Virus replication kinetics in HNE culture. Apical washes were collected daily from the other three HNE (*n* = 3) cultures infected by each recombinant virus. Viral titer in apical washes was determined by plaque assay in Vero E6-TMPRSS2-T2A-ACE2 cells. Viral titers were the average of 3 transwells ± standard deviation. Data were analyzed using two-way ANOVA and one-way ANOVA (*P < 0.05; ***P < 0.001; ****P < 0.001). The *P* values shown were 0.011 and <0.0001.

**Fig 13:**
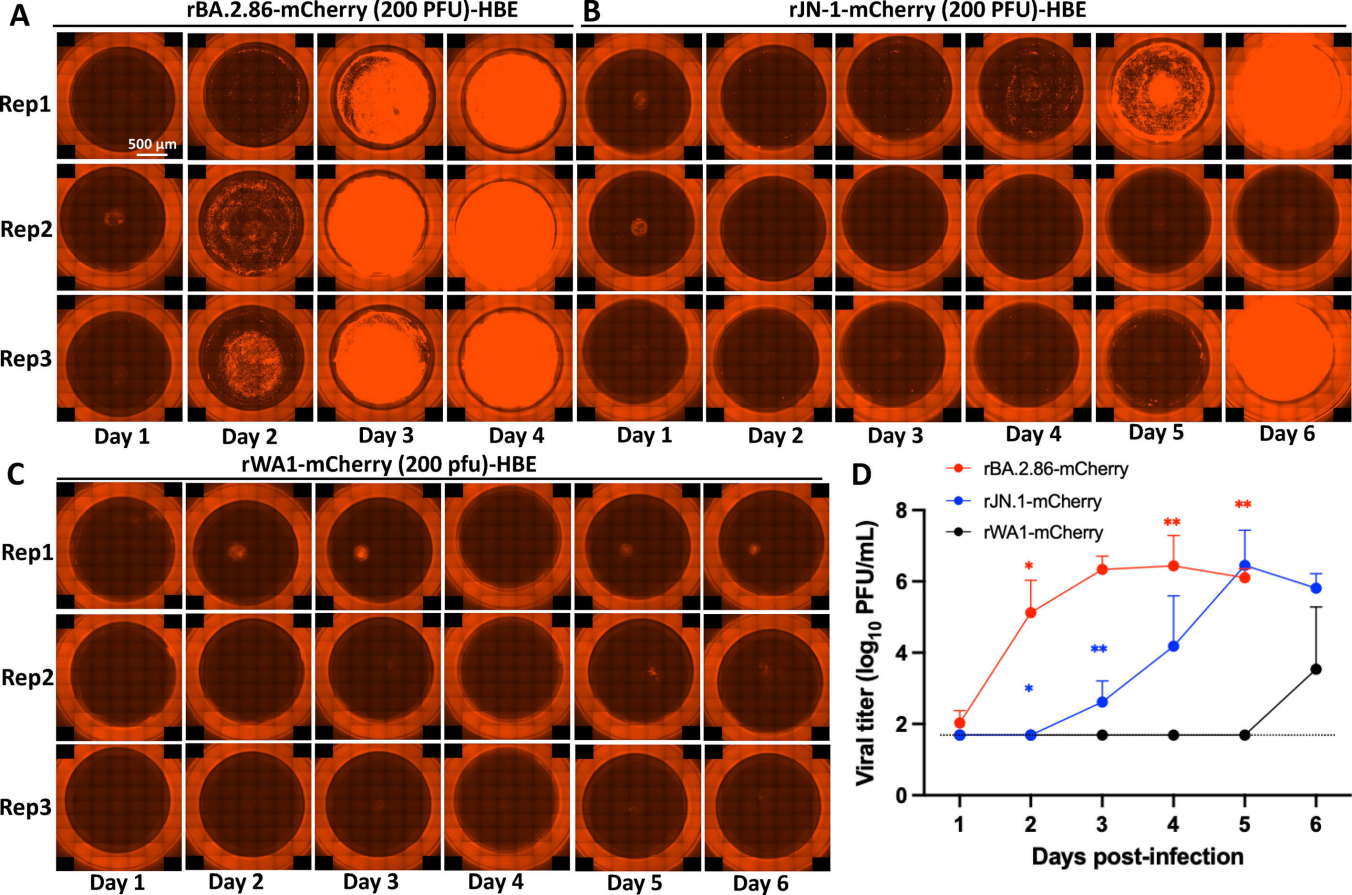
Spread and replication of rBA.2.86-mCherry, rJN.1-mCherry, and rWA1-mCherry in HBE cultures at 200 PFU. HBE (*n* = 6) cultures from donor 3 were grown in 24-well transwell plates and were inoculated with 200 PFU of rBA.2.86-mCherry (**A**), rJN.1-mCherry (**B**), or rWA1-mCherry (**C**). Three HBE (*n* = 3) cultures were used for monitoring the spread of the mCherry signal daily by fluorescence microscopy until the mCherry had spread to the entire transwell and scanned by Image J. Scale bar for all transwell images is 500 μm. (**D**) Virus replication kinetics in HBE cultures. Apical washes were collected daily from the other three HBE (*n* = 3) cultures infected by each recombinant virus. Viral titer in apical washes was determined by plaque assay in Vero E6-TMPRSS2-T2A-ACE2 cells. Viral titers were the average of 3 transwells ± standard deviation. Data were analyzed using two-way ANOVA and one-way ANOVA (*P < 0.05; **P < 0.01). The red stars indicate the statistical differences of rBA.2.86-mCherry compared to the rWA1-mCherry (the *P* values shown were 0.0227, 0.0022, 0.0032, and 0.0011). The blue stars indicate the statistical differences of rJN1-mCherry compared to the rBA.2.86-mCherry (the *P* values shown were 0.0227 and 0.0016).

Similar results were observed when HNE (Fig. 12) and HBE (Fig. 13) cultures (from donor 3) were inoculated with 200 PFU of rBA.2.86-mCherry, rJN.1-mCherry, or rWA1-mCherry. rBA.2.86-mCherry had visible mCherry expression at day 1, increased mCherry signal at day 2, and the entire transwell had turned red by day 3 (Fig. 12A). rJN.1-mCherry produced visible mCherry-positive cells at day 1, gradually increased by day 2, and reached a peak that involved most of the cultures by day 4 (Fig. 12B). The titer of the virus released from the apical surface of rBA.2.86-mCherry–infected HNE cultures was higher than that of the rJN.1-mCherry–infected HNE cultures (Fig. 12C). The difference in viral spread and replication of rBA.2.86-mCherry and rJN.1-mCherry in HBE cultures (Fig. 13) was larger than that in HNE cultures (Fig. 12). The mCherry signal of rBA.2.86-mCherry spread to the entire transwell at day 3 (Fig. 13A), whereas only two out of three transwells inoculated with rJN.1-mCherry had a robust mCherry signal by day 6. The third transwell had little mCherry expression at day 6 (Fig. 13B). rBA.2.86-mCherry had robust virus replication and reached a peak titer at day 3, whereas replication of rJN.1-mCherry was significantly delayed, reaching its peak titer at day 5 (Fig. 13C). These results confirmed that rBA.2.86-mCherry replicates faster and spreads more robustly than rJN.1-mCherry in both HNE and HBE cultures derived from three donors using different doses of virus.

## DISCUSSION

CoVs contain one of the largest RNA genomes (26 to 32 kb) among known RNA viruses. The development of reverse genetics systems for CoVs has been highly challenging due to the toxicity or the instability of viral cDNA in bacterial systems often resulting in unwanted deletions or mutations during the cloning process. In this study, we developed a yeast-based recombinant system for the rapid construction of infectious cDNA clones for SARS-CoV-2. This method is simple, rapid, accurate, and highly efficient. The entire protocol from assembly of the full-length genome to virus recovery can be performed in 2 to 3 weeks. To our knowledge, this is the first reverse genetics approach for rescuing SARS-CoV-2 based on yeast ligation and transfection of a single BAC plasmid. By leveraging the homologous recombination capabilities of Saccharomyces *cerevisiae*, we have been able to seamlessly assemble the full-length SARS-CoV-2 genome—including mutations, deletions, or insertions, and even an additional reporter gene—in a single step and without using *in vitro* restriction enzymes and ligases. Our system uses a CMV promoter to drive the transcription of the viral genome RNA directly in transfected cells, avoiding the need for *in vitro* RNA synthesis, thereby reducing the technical complexity and improving the rescue efficiency.

The steps and key technical elements in our protocol are summarized in Fig. 1A.

### Step 1: cDNA preparation

DNA fragments can be commercially synthesized quickly or produced by RT-PCR from the viral RNA genome. These fragments require 30–50 nucleotide (nt) overhangs to link them to neighboring fragments. To minimize mutations at this step, we generated fragments of less than 5 kb by PCR using high-fidelity PCR enzymes. The overhang sequences in the cDNAs should avoid using self-complementary sequences, repetitive DNA sequences, internal repeats, and sequences with high secondary structures, which could prevent self-ligation and off-target recombination events.

### Step 2: yeast ligation

Mix the DNA fragments and transform yeast cells with the DNA. The yeast assembles the full-length SARS-CoV-2 genome in BAC plasmid in a single step. We use 100 ng of each DNA fragment for yeast transformation. If no or very few yeast colonies grow on the plates, increase the mixture to 200 ng of each DNA fragment for yeast transformation.

### Step 3: large-scale plasmid preparation

Because the concentration of the plasmid extracted from yeast cells is low, the plasmid should be electroporated into DH10B competent cells to produce a midiprep or maxiprep. If no bacterial colonies grow in the plate, increase the amount of yeast plasmid electroporated.

### Step 4: virus recovery

Transfect the plasmid into susceptible cells for virus rescue. We use Vero E6-TMPRSS2-T2A-ACE2 cells as they are very sensitive to SARS-CoV-2 replication and spread, increasing the recovery efficiency and minimizing the possibility of losing the furin cleavage site in the spike protein.

Prior to the COVID-19 pandemic, BAC plasmid (DNA)-launched reverse genetics system and *in vitro* ligation/T7 transcription (RNA)-launch reverse genetics systems were the two most common methods for rescuing CoVs (16). The BAC-based system integrates the entire viral genome into a single BAC plasmid, enabling stable propagation in bacterial cells. Unlike most plasmids, BACs replicate at a low copy number (one or two copies) per bacterial cell, reducing the likelihood of mutations/instability and toxicity of the inserted DNA. This approach was first used for the construction of TGEV infectious clones in 2000 (18) and has been used for the construction of several other porcine coronaviruses (36, 37). However, the traditional BAC-based approach involves cloning each cDNA fragment sequentially into a BAC vector using naturally occurring unique restriction enzyme sites and/or artificially creating restriction enzyme sites in the CoV genome. These multiple rounds of restriction enzyme digestion and ligation of cDNA to BAC vector are time-consuming, labor-intensive, and technically demanding. In the BAC plasmid, the full-length CoV genome is under the control of the CMV promoter at the 5′ end and is flanked at its terminus by poly(A) sequences at the 3′ end, followed by the HDVRz and bGH termination sequences. Following the transfection of the plasmid into susceptible cells, the cDNA is transcribed within the nucleus, and the RNA is subsequently translocated to the cytoplasm, leading to the efficient recovery of recombinant virus. The traditional BAC-based approach has been used for the construction of SARS-CoV-2 WA1 infectious clones and has become one of the most popular methods for the manipulation of the SARS-CoV-2 genome (24, 38, 39).

Unlike the traditional BAC approach, our yeast-based recombination method assembles the SARS-CoV-2 genome into the BAC vector in a single step. This is achieved by engineering a yeast origin of replication into the BAC vector, allowing the plasmid to assemble and replicate in yeast cells. Our method is superior to the traditional BAC-based method by eliminating the mutations neeed to insert restriction sites and the labor-intensive and time-consuming cloning steps and thereby minimizing the potential of mutations during multiple rounds of transformation into competent bacterial cells. Using this novel method, we recently constructed a panel of SARS-CoV-2 live-attenuated vaccine candidates and live-attenuated SARS-CoV-2-vectored human respiratory syncytial virus vaccine candidates (40).

The next most commonly used approach is the *in vitro* ligation/T7 transcription method, which assembles the full-length CoV genomic cDNA under the control of a T7 promoter through *in vitro* ligation, transcribes the full-length viral RNA genome using a T7 RNA polymerase, and is followed by electroporation of the RNA into susceptible cells for virus recovery (22). Although it avoids the toxicity issues associated with bacterial cloning, it is also labor-intensive and time-consuming, requiring the preparation of subclones encoding different cDNA fragments and multiple ligation steps. Additionally, *in vitro* transcription of 5′-capped and methylated, and 3′-polyadenylated 30 kb CoV genome may be inefficient, and electroporation of RNA into susceptible cells sometimes results in low recovery efficiency (19, 22, 23). This method has been successfully used for constructing infectious clones for TGEV (22), SARS-CoV (20), MERS-CoV (21), SARS-CoV-2 (19), and pangolin CoV (41).

During the pandemic, several other techniques emerged to enable SARS-CoV-2 reverse genetics. Transformation-associated recombination (TAR) cloning in yeast artificial chromosomes (YACs) leverages the high tolerance of *Saccharomyces cerevisiae* to toxic viral sequences and its efficient homologous recombination machinery (25). Although it allows for the assembly of the full-length SARS-CoV-2 genome in a single step, it requires additional steps, including *in vitro* transcription of the entire viral genome and electroporation of RNA to the host cells, which can limit viral recovery efficiency (25). In addition, three bacterium- and yeast-free reverse genetics systems were developed for SARS-CoV-2, all of which were driven by a CMV promoter. Circular polymerase extension reaction (CPER) assembles overlapping DNA fragments into circular DNA in a plasmid or linker, followed by transfection into susceptible cells (26), whereas “infectious subgenomic amplicons” (ISA) (27) and “cloning-free and exchangeable system for virus engineering and rescue” (CLEVER) (an improved version of ISA) (28) methods directly transfect overlapping DNA fragments to the eukaryotic cells for recombination into a full-length genome cDNA copy and virus recovery, without the need for assembly into a full-length genome cDNA copy *in vitro*. While each method has its own advantages, the plasmid-based approach is generally easier for manipulation, distribution, and storage. Our yeast-based approach has many unique advantages, including rapidity, high efficiency, simplicity, and adaptability to other coronaviruses and RNA viruses.

Reporter viruses are useful for monitoring virus infection and spread *in vitro* and *in vivo*, for studying the virus, and for developing countermeasures. Using our rapid yeast-based platform, we have in this study generated reverse genetic systems for SARS-CoV-2 WA1, BA.2.86, and JN.1 viruses expressing mCherry, GFP, or Nluc reporter viruses. These rSARS-CoV-2 reporter viruses exhibited replication kinetics and virus yield in Vero E6-TMPRSS2-T2A-ACE2 cells that were indistinguishable from their parental viruses, indicating that insertion of these reporter genes does not significantly disturb virus replication. All these reporter genes were fused to the viral N gene connected by the P2A sequence, which induces ribosomal skipping during translation, allowing high-level expression of reporter genes without affecting viral replication.

At the beginning of the pandemic, several groups generated rSARS-CoV-2 WA1 expressing reporters such as mNeonGreen (mNG), GFP, GFP-fused nanoluciferase (Nluc), and mCherry, in which viral ORF7a was replaced by the reporter gene (19, 23, 24)—an approach that had previously been used to generate SARS-CoV expressing a reporter (20). However, it has been shown that replacement of ORF7a with the reporter gene resulted in low levels of reporter gene expression, making it difficult to track virus infection in cell cultures and in animals using *in vivo* imaging systems (IVIS) (39). Using SARS-CoV-2 WA1 as the backbone, Ye et al. reported that the expression level of reporter genes (Venus fluorescent protein or Nano luciferase) was dramatically increased by fusing them with the viral N gene, allowing for monitoring virus infection *in vitro* and *in vivo* using IVIS in a real-time manner (38, 39). In addition, the resultant reporter viruses rSARS-CoV-2/Venus-2A and rSARS-CoV-2/Nluc-2A exhibited high stability in cell culture and retained wild-type (WT) virus-like pathogenicity *in vivo* (38). Therefore, a recombinant virus with a reporter fused with the viral N protein gene better mimics WT virus infection compared to the ORF7a-deleted reporter viruses. The N gene-fused reporter virus is likely a better tool to investigate viral infection, dissemination, and pathogenesis, and to evaluate therapeutic interventions.

The emergence of the Omicron subvariant BA.2.86 was a turning point for SARS-CoV-2 evolution during the pandemic. BA.2.86 is derived from the BA.2 subvariant but carries over 30 mutations in its spike protein relative to the ancestral BA.2 virus and up to 35 mutations relative to previously dominant subvariant XBB.1.5. Interestingly, BA.2.86 did not become a dominant virus. However, JN.1, which only differs from BA.2.86 by four amino acids, did become the dominant virus. Among these four amino acid differences, only one (L455S) is located in the spike. The other three substitutions, T1892A, R249K, and F19L, are located in the viral nsp3, nsp6, and ORF7b proteins, respectively. Several studies have provided some mechanistic insights into why JN.1, rather than BA.2.86, became a dominant virus. It was shown that the EC_50_ of hACE2 binding by the JN.1 spike was enhanced by 1.8-fold relative to the BA.2.86 spike (10, 14), indicating that the JN.1 spike has a decreased affinity for binding to hACE2 receptor, which may enhance virus transmissibility. Structural analysis showed that L455 is located on the binding interface between hACE2 and the receptor binding domain of the spike. Therefore, L455S likely reduced the binding affinity between hACE2 and the receptor binding domain of the JN.1 spike. Using pseudotype neutralization assays, many studies have shown that JN.1 exhibits significantly greater neutralizing antibody escape and immune evasion compared to BA.2.86 (8–10, 12). However, little information is available for comparing the replication and spread of Omicron BA.2.86 and JN.1 in *ex vivo* human nasal and lung epithelial cultures. To date, only one study has examined viral RNA release from BA.2.86- and JN.1-infected HNE culture. Specifically, viral RNA release (measured by RT-qPCR) from the apical side of HNE from Omicron BA.2.86 was slightly higher than that from JN.1 when HNE cultures were infected with 2,000 infectious units of each virus (10). However, virus titers and the ability of these viruses to spread in HNE culture were not determined in their study (10).

In this study, we directly compared the replication and spread of SARS-CoV-2 WA1, BA.2.86, and JN.1 in *ex vivo* nasal and lung epithelial cultures derived from three different donors using mCherry reporter viruses. Interestingly, these viruses were grown and titrated on Vero E6-TMPRSS2-T2A-ACE2 cells, but they varied widely in their infectivity for HBE and HNE cells. As expected, both BA.2.86 and JN.1 infected, replicated, and spread much faster than the ancestral SARS-CoV-2 WA1 in HNE and HBE cultures. This is consistent with a previous observation that the Omicron variant replicates faster than SARS-CoV-2 WA1 (30). Another interesting observation is that all three viruses replicated and spread faster in HNE cultures compared to HBE cultures. This is consistent with the observation that the ACE2 receptor has the highest expression in the nose and its expression decreases throughout the lower respiratory tract (19).

Surprisingly, BA.2.86 replicated and spread faster than JN.1 in HNE and HBE cultures at all four levels of inoculation (100, 200, 500, and 1,000 PFU). BA.2.86 had peak titer and spread throughout the entire transwell by 3 dpi, whereas JN.1 exhibited a significant delay, reaching its peak titer at 5 dpi. In addition, infectious virus released from the apical surface of BA.2.86-infected HNE and HBE cultures was higher than those released from JN.1-infected HNE and HBE cultures. Multiple factors may collectively contribute to the dominance of a variant in the human population. Although JN.1 displays a relative decrease in viral replication and spread in HNE and HBE cultures compared to BA.2.86, its enhanced immune evasion and decreased hACE2 binding affinity mediated by the L455S mutation in the JN.1 spike appears to enable JN.1 to outcompete its rival, BA.2.86, becoming a dominant virus. Because mutations in non-spike proteins may affect viral replication, future studies will be needed to determine whether L455S alone or the other three mutations in non-spike proteins collectively contributed to the reduced spread and replication of JN.1 in HNE and HBE cultures.

In summary, we developed a powerful and convenient method that can rapidly modify the SARS-CoV-2 genome. This system could be an important tool for understanding SARS-CoV-2 biology and for the rational design of live-attenuated SARS-CoV-2 vaccines. Furthermore, the yeast-based BAC system described in our study can be rapidly adapted for other large RNA viruses, providing a versatile tool for establishing reverse genetics systems across a wide range of viral pathogens.

## MATERIALS AND METHODS

### Cells and viruses

The IVSSc1 S. *cerevisiae* yeast strain (Invitrogen, Cat. No. C81000) and MegaX DH10B T1R Electrocomp Cells (Invitrogen, Cat. No. C640003) were used for ligation and cloning processes. Vero E6 (ATCC No. CRL-1586) and Vero E6-TMPRSS2-T2A-ACE2 (BEI, NR-54970) were grown in Dulbecco’s modified Eagle’s medium (DMEM) containing 4 mM L-glutamine, 4,500 mg/L glucose, 1 mM sodium pyruvate, and 1,500 mg/L sodium bicarbonate, supplemented with 10% fetal bovine serum. Vero E6-TMPRSS2-T2A-ACE2 cells were additionally maintained in medium containing 10 μg/mL puromycin. The SARS-CoV-2 USA-WA1/2020 natural isolate (SARS-CoV-2 WA, NR-52281), SARS-CoV-2 BA.2.86 (NR-59638), and SARS-CoV-2 JN.1 (NR-59693) were obtained from BEI Resources. All three SARS-CoV-2 strains were propagated and titrated on Vero E6-TMPRSS2-T2A-ACE2 cells.

### Construction of BAC plasmids encoding full-length genome of SARS-CoV-2 (pSMART-SARS-CoV-2)

The full-length genomic cDNA of SARS-CoV-2 was cloned into the bacterial artificial chromosome (BAC) backbone vector, pSMART-BAC, which was engineered to include a yeast replication origin, a URA3 promoter, and a URA3 yeast selection marker derived from the plasmid pYES1L. The SARS-CoV-2 genome was placed under the control of the cytomegalovirus (CMV) promoter at the 5′ end, with its 3′ termini flanked by 30 nt of polyadenylation sequences, the hepatitis delta virus ribozyme (HDVRz), and the bovine growth hormone (bGH) termination. The full-length cDNA clone of SARS-CoV-2 was assembled using seven overlapping fragments along with an additional eighth fragment containing a reporter gene (such as mCherry, GFP, or Nluc). The seven overlapping fragments, ranging from 1,605 bp to 6,239 bp (F1 to F7), were amplified from SARS-CoV-2 RNA by RT-PCR. The pSMART-BAC vector was split into two overlapping fragments (V1 and V2) and was amplified by PCR. All DNA fragments were purified using a PCR purification kit (QIAquick PCR Purification Kit, Qiagen). The assembly was performed using a yeast recombination system. Specifically, 100 ng of the pSMART-BAC vector was combined with 100 ng of each SARS-CoV-2 fragment and reporter gene fragment in a polyethylene glycol (PEG)/lithium acetate (LiAc) solution. The mixture was transformed into *Saccharomyces cerevisiae* yeast competent cells via heat shock and then plated onto SD/Ura⁻ agar plates. After a 2-day incubation at 30°C, individual yeast colonies were picked and cultured overnight in SD/Ura⁻ broth at 30°C. Plasmid DNA was extracted from yeast cells and used for PCR screening by targeting the F3 and F5 fragments. Positive plasmids were then electroporated into MegaX DH10B competent cells and grown on LB plates with chloramphenicol. Three bacterial colonies from each yeast plasmid positive for pSMART-SARS-CoV-2 DNA were picked from LB plates and were inoculated into separate culture tubes containing 2 mL of LB medium with chloramphenicol. The culture was incubated at 30°C with shaking at 220 rpm for 24 h. The plasmid DNA was extracted using the Qiagen plasmid DNA extraction kit and screened by PCR amplifying the F3 and F5 fragments. Subsequently, the plasmid DNA was digested by HindIII and visualized in agarose gel. Finally, the plasmids were sequenced by the Plasmidsaurus (Louisville, KY). Correct plasmids were used for transfections for virus rescue.

### Rescue of rSARS-CoV-2

Vero E6-TMPRSS2-T2A-ACE2 cells at 80–90% confluence in a 12-well plate were transfected with 2 µg of pSMART-BAC-SARS-CoV-2 plasmid DNA using Lipofectamine 2000 in 250 μL of Opti-MEM. Twenty-four hours post-transfection, the medium was replaced with 2 mL of post-infection medium (DMEM supplemented with 2% FBS). The cells were monitored daily for cytopathic effects (CPE). At 72 h post-transfection, the supernatant from the transfected Vero E6-TMPRSS2-T2A-ACE2 cells was collected, and 100 μL was used to infect fresh Vero E6-TMPRSS2-T2A-ACE2 cells in a T25 flask. After extensive CPE was observed, supernatant was collected, aliquoted, and stored in a −80 °C freezer. Total RNA was extracted from 200 μL of supernatant using the TRIzol method (Invitrogen). Virus rescue was confirmed by RT-PCR and sequencing. Viral titer was determined by plaque assay.

### Virus growth curve

Vero E6-TMPRSS2-T2A-ACE2 cells were inoculated with each recombinant virus at an MOI of 0.01. After 1 h, the inoculum was removed, the cells were washed twice with DMEM, fresh DMEM (supplemented with 2% FBS) was added, and the infected cells were incubated at 37°C. At the indicated time, photographs of CPEs were captured using a light microscope, and mCherry and GFP expression was visualized with a fluorescent microscope. The cell culture supernatant was harvested at the indicated time points, and virus titers were determined by plaque assay in Vero E6-TMPRSS2-T2A-ACE2 cells.

### Plaque assay

Plaque assay was carried out using the protocol published previously (42–45). Confluent Vero E6-TMPRSS2-T2A-ACE2 monolayer cells in 12-well plates were inoculated with 10-fold serial dilutions of virus. After adsorption for 1 h at 37°C, cells were overlaid with 1 mL of DMEM containing 0.25% (wt/vol) low-melting temperature agarose, 0.12% (vol/vol) NaHCO_3_, 2% (vol/vol) FBS, 25 mM HEPES, 2 mM L-glutamine, 100 µg/mL of streptomycin, and 100 U/mL penicillin. After incubation at 37°C for 2 days, cells were fixed with 4% paraformaldehyde for 1 h. After fixation, the overlay was discarded, and viral plaques were visualized by staining with 0.05% (vol/vol) crystal violet.

### RT-PCR and sequencing

All PCR products, plasmids, and viral stocks were sequenced to confirm their identity. Viral RNA was extracted from 100 µL of each recombinant virus using the TRIzol method (Invitrogen). Eight overlapping DNA fragments spanning the SARS-CoV-2 genome were amplified by RT-PCR. The PCR products were purified and sequenced using sequencing primers at The Ohio State University Plant Microbe Genetics Facility or Plasmidsaurus (Louisville, KY).

### Viral infection in a primary human nasal and airway cell culture model

Well-differentiated primary human nasal epithelial (HNE) and primary human bronchial epithelial (HBE) cultures were prepared as described in our previous publications (46–48). Briefly, the HNE and HBE progenitor cells were isolated from the internal lining of nasal and bronchial tissue, respectively, from a healthy human donor (under IRB waiver MOD00014135) seeded on collagen-coated transwells and differentiated at the air-liquid interface (ALI). After maturation at week 4, the HNE and HBE cultures were ready for virus infection. Viral titer used for infection was determined in Vero E6-TMPRSS2-T2A-ACE2 cells. Prior to inoculation, the apical surface of the HNE or HBE culture in transwells was washed five times with 100 μL of pre-warm DMEM, and the basal medium was refreshed. This step is essential for virus infection because the washing step removes much of the mucus coating the apical surface. Three independent infection experiments were conducted in HNE and HBE cultures derived from three different donors.

#### Experiment 1

HNE (*n* = 4) and HBE (*n* = 4) cultures in transwells were inoculated with 100 μL of DMEM containing 100 or 500 PFU of rBA.2.86-mCherry, rJN.1-mCherry, or rWA1-mCherry, which was equivalent to an MOI of 1.67 × 10^−4^ or 8.33 × 10^−4^. Four extra HNE (*n* = 4) and HBE (*n* = 4) transwells were inoculated with 10,000 PFU of rWA1-mCherry, which was equivalent to an MOI of 1.67 × 10^−2^. After virus inoculation, the plates were incubated at 37°C with 5% CO_2_, with gentle shaking by hand every 10 min. Following a 2-hour incubation, the inoculum was removed, and the culture was washed three times with PBS. The infected HNE and HBE cultures were maintained without medium in the apical compartment, while fresh medium was supplied through the basal compartment. Three hundred microliters of PBS was added to the apical side of the HNE or HBE culture from days 2 to 3 post-infection and incubated at 37°C for 30 min to elute the released viruses. Virus yield in the apical wash was determined by plaque assay on Vero E6-TMPRSS2-T2A-ACE2 cells. After washing, the transwells were fixed in 10% formalin for 2h. The mCherry expression was visualized by a fluorescence microscope, and the entire transwell scanned using the Image J software.

#### Experiment 2

The HNE and HBE cultures were derived from a different donor (donor 2). The HNE (*n* = 8) and HBE (*n* = 8) cultures were inoculated with 100 µL of DMEM containing 1,000 PFU (equivalent to MOI of 1.67 × 10^−3^) of rBA.2.86-mCherry or rJN.1-mCherry. The procedure for virus infection, imaging, and collection of apical washes was identical to Experiment 1. At each day (from days 1 to 5), one transwell was fixed by formalin and subsequently scanned using Image J. The other three transwells were used for examination of viral replication. At each day (from days 1 to 5), apical washes of these three HNE and HBE cultures were collected for virus titration by plaque assay on Vero E6-TMPRSS2-T2A-ACE2 cells.

#### Experiment 3

HNE (*n* = 6) and HBE (*n* = 6) cultures derived from a third donor were grown in transwells in 24-well plates. There were some adjustments during virus infection compared to experiments 1 and 2. After removing the mucus on the apical surface, HNE and HBE cultures were inoculated with 50 μL of DMEM containing 1,000 PFU (MOI of 1.67 × 10^−3^) or 200 PFU (MOI of 3.33 × 10^−4^) of each virus. Then, the plates were placed on a rocker with constant rocking in a 37°C incubator. After a 2-hour incubation and rocking, the inoculum was removed, and the culture was washed three times with PBS. Among these six transwells, three transwells infected by each virus were used for monitoring virus spread, and the other three transwells infected by the same virus were washed daily for determination of viral titer. In this experiment, the plates were not fixed by 10% formalin. Instead, each day, the mCherry expression in all plates was visualized by a fluorescence microscope and scanned using the Image J software. After imaging, the plates were returned to the 37°C incubator. The dynamics of mCherry spread in the same transwell was recorded daily. For the other three transwells, 300 μL of PBS was added to the apical side of the HNE or HBE cultures daily and incubated at 37°C for 30 min to elute the released viruses, which were titrated by plaque assay on Vero E6-TMPRSS2-T2A-ACE2 cells.

### Statistical analysis

Statistical analysis was performed using Student’s *t*-test, one-way or two-way ANOVA with multiple comparisons in GraphPad Prism software (San Diego, CA). A *P* value of <0.05 was considered statistically significant.

## Data Availability

All data are provided in the figures. The sequences of recombinant SARS-CoV-2 reporter viruses are available in GenBank using the following accession numbers: rSARS-CoV-2-WA1-mCherry (PX943830), rSARS-CoV-2-WA1-GFP (PX943831), rSARS-CoV-2-WA1-Nluc (PX943832), rSARS-CoV-2-BA.2.86-mCherry (PX943833), rSARS-CoV-2-BA.2.86-Nluc (PX943834), rSARS-CoV-2-BA.2.86-GFP (PX943835), rSARS-CoV-2-JN.1-mCherry (PX943836), rSARS-CoV-2-JN.1-GFP (PX943837), and rSARS-CoV-2-JN.1-Nluc (PX943838).
